# Inherited Arrhythmias in the Pediatric Population: An Updated Overview

**DOI:** 10.3390/medicina60010094

**Published:** 2024-01-03

**Authors:** Marco Valerio Mariani, Nicola Pierucci, Francesca Fanisio, Domenico Laviola, Giacomo Silvetti, Agostino Piro, Vincenzo Mirco La Fazia, Cristina Chimenti, Marco Rebecchi, Fabrizio Drago, Fabio Miraldi, Andrea Natale, Carmine Dario Vizza, Carlo Lavalle

**Affiliations:** 1 Department of Cardiovascular, Respiratory, Nephrological, Aenesthesiological and Geriatric Sciences, “Sapienza” University of Rome, 00161 Rome, Italy; nicola.pierucci@uniroma1.it (N.P.); domenico.laviola@uniroma1.it (D.L.); giacomo.silvetti@uniroma1.it (G.S.); agostino.piro@uniroma1.it (A.P.); cristina.chimenti@uniroma1.it (C.C.); dario.vizza@uniroma1.it (C.D.V.); carlolavalle@yahoo.it (C.L.); 2 Division of Cardiology, Policlinico Casilino, 00169 Rome, Italy; fanisio.francesca@gmail.com (F.F.); marcorebecchi3@virgilio.it (M.R.); 3 Department of Electrophysiology, St. David’s Medical Center, Texas Cardiac Arrhythmia Institute, Austin, TX 78705, USA; vmirco.lafazia@gmail.com (V.M.L.F.); dr.natale@gmail.com (A.N.); 4 Department of Pediatric Cardiology and Cardiac Surgery, Bambino Gesù Children’s Hospital and Research Institute, 00165 Rome, Italy; fabrizio.drago@opbg.net; 5 Cardio Thoracic-Vascular and Organ Transplantation Surgery Department, Policlinico Umberto I Hospital, 00161 Rome, Italy; fabio.miraldi@uniroma1.it

**Keywords:** inherited arrhythmias, pediatric cardiomyopathies, sudden cardiac death

## Abstract

Pediatric cardiomyopathies (CMs) and electrical diseases constitute a heterogeneous spectrum of disorders distinguished by structural and electrical abnormalities in the heart muscle, attributed to a genetic variant. They rank among the main causes of morbidity and mortality in the pediatric population, with an annual incidence of 1.1–1.5 per 100,000 in children under the age of 18. The most common conditions are dilated cardiomyopathy (DCM) and hypertrophic cardiomyopathy (HCM). Despite great enthusiasm for research in this field, studies in this population are still limited, and the management and treatment often follow adult recommendations, which have significantly more data on treatment benefits. Although adult and pediatric cardiac diseases share similar morphological and clinical manifestations, their outcomes significantly differ. This review summarizes the latest evidence on genetics, clinical characteristics, management, and updated outcomes of primary pediatric CMs and electrical diseases, including DCM, HCM, arrhythmogenic right ventricular cardiomyopathy (ARVC), Brugada syndrome (BrS), catecholaminergic polymorphic ventricular tachycardia (CPVT), long QT syndrome (LQTS), and short QT syndrome (SQTS).

## 1. Introduction

The significance of inheritance in arrhythmias in the pediatric population has been extensively demonstrated in both genetic and epidemiological studies [1].

The role of genetics in inherited arrhythmias (IAs) is crucial both in arrhythmic conditions, which occur in hearts with macroscopic structural heart disease (SHD), and in apparently non-structural heart conditions known as channelopathies [2,3].

Considering the definition of cardiomyopathy (CM) as a disease of the myocardium associated with cardiac dysfunction (World Heart Organization 1995) [4], Corrado et al. in 2005 [2] emphasized the need to include non-structural inherited arrhythmic conditions as CMs. The Padua group stressed the need to introduce a genomic classification of arrhythmogenic heart diseases that does not consider only macroscopic SHD [3]. As a matter of fact, in so-called non-structural conditions (long QT syndrome [LQTS] and short QT syndrome [SQTS], Brugada syndrome [BrS], Lenegre disease, catecholaminergic polymorphic ventricular tachycardia [CPVT]), the myocyte is abnormal, although the heart is apparently intact.

Recent European Society of Cardiology (ESC) guidelines on CM together with the European Heart Rhythm Association/Heart Rhythm Society/Latin American Heart Rhythm Society (EHRA/HRS/LAHRS) Expert Consensus Statement highlighted a lack of evidence to include channelopathies in the group of CMs [5]. However, it is essential to acknowledge the role of genes encoding for ion channels in the diagnostic definition and prognostic stratification of patients with dilated and non-dilated cardiomyopathy, conduction disorders, and arrhythmias [6].

**Figure 1:**
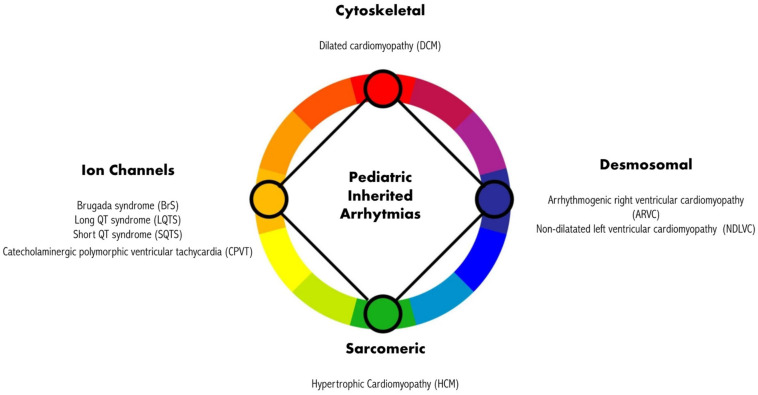
Mutations in genes encoding different proteins are responsible for different inherited cardiomyopathies and related arrhythmic manifestations.

The pivotal role of genetics, beyond the definition of CM, highlights that IA can arise from mutations in genes that primarily encode four main types of protein, as shown in Figure 1 [1]. These proteins can be classified as sarcomeric, which predominantly causes hypertrophic cardiomyopathy (HCM); cytoskeletal, which mainly causes dilated cardiomyopathy (DCM); desmosomal, which primarily causes arrhythmogenic right ventricular cardiomyopathy (ARVC); and non-dilatated left ventricular cardiomyopathy (NDLVC) and ion channels, which give rise to electrical diseases (or channelopathies). This latter group encompasses BrS, LQTS, SQTS, CPVT, and atrial fibrillation (AF). A number of these conditions in the pediatric population are highly fatal, even in the early stages [7,8,9,10,11].

Moreover, it should be considered that many of these conditions exhibit a significant degree of genetic and allelic heterogeneity, meaning that numerous variants in various genes can lead to the same phenotype. Pathogenic variants that are rare and linked to cardiomyopathies frequently display incomplete and age-related penetrance, as well as variable expressivity [12,13].

The utilization of next-generation sequencing (NGS) techniques, by allowing for the simultaneous analysis of multiple genes, enables the detection of pathogenic mutations associated with cardiomyopathies and channelopathies in over 200 distinct genes. With the inclusion of genes previously considered rare for clinical manifestations in molecular testing, the rate of identifying disease-causing variants has significantly risen. However, there are a number of variants that are currently of uncertain significance (VUSs), and more studies are needed to determine the pathogenic role of VUSs not only in phenotype-positive patients but also in asymptomatic and phenotype-negative individuals [14].

## 2. Cardiomyopathies

Pediatric CMs are rare conditions characterized by an annual incidence of 1.1–1.5/100.000 in children below age 18 [13,14,15].

Data about CMs are primarily derived from large international registries and single-center studies. Consistent evidence on the risk factors for sudden cardiac death (SCD) and prevention strategies along with disease-specific therapies is still lacking, and larger studies are needed [15,16,17,18,19,20].

The most common conditions in the North America Pediatric CM Registry (PCMR, 1994) were DCM and HCM, characterized by an annual incidence of 0.57 and 0.47/100.000 [16,21,22,23,24].

Historically, arrhythmogenic cardiomyopathies (ACMs) have been described in young adults; however, a growing number of children have been diagnosed with the condition [25].

Although clinical and imaging assessment may be more difficult in children, early detection of signs and symptoms indicative of secondary CM that may necessitate specific treatment is of paramount importance.

According to the ESC 2023 guidelines, the definition of CM includes myocardial disorder, in which the heart muscle is structurally and functionally abnormal, in the absence of coronary artery disease (CAD), hypertension, valvular heart disease (VHD), and congenital heart disease (CHD) sufficient to cause the observed myocardial modifications [6].

This definition can be applied to both children and adults and should be primarily focused on the phenotype rather than on myocardial pathology and etiology.

One of the most important changes in recent ESC guidelines is related to the term ACM, which includes a group of conditions characterized by structural and functional abnormalities of the myocardium associated with ventricular arrhythmia (VA) [6]. Given the clinical and genetic overlap between right ventricular (RV) and left ventricular (LV) CMs, the use of the term ACM has been broadly used in clinical practice. However, since there is no widely agreed-upon definition of this term, recent guidelines suggest considering “ACM” as a blanket designation.

Indeed, ACM is now used to refer to a wide variety of different conditions, which can lead to inconsistencies and contradictions when used in a clinical context [26].

The task force emphasizes the critical role of arrhythmia as a diagnostic warning sign and prognostic marker across a range of clinical phenotypes, but it does not recommend the use of the term ACM as a specific CM subtype because it lacks a morphological, etiological, and functional definition [27,28].

CM accounts for life-threatening arrhythmogenic disorders in the pediatric population [1,2,3,4,5,6,7,8]. However clinical presentation and outcomes of CM that manifest in infants (<1 year of age) can differ significantly from those observed in older children, adolescents, and adults. In cases of infantile and early childhood-onset CM, management depends on clinical presentation, cardiac phenotype, and etiology [29]. Severe clinical onset is typically managed in intensive or subintensive care units by neonatologists and pediatric cardiologists, who address respiratory distress, metabolic acidosis, hypoglycemia, and hypotonia [30,31]. A comprehensive clinical approach that considers both cardiac and systemic phenotypes (including dysmorphisms, consanguinity, skeletal anomalies, mental retardation, muscle hypotonia and weakness, hypoglycemia, aciduria, free fatty acid profiles, and calcium and vitamin D metabolism) is essential to guide management when reversible or specific diseases are present [32]. This approach should involve a multidisciplinary team that includes geneticists and experts in metabolic and neurological diseases.

## 3. Arrhythmogenic Right Ventricular Cardiomyopathy (ARVC)

### 3.1. Introduction

ARVC is an inherited disorder characterized by progressive heart muscle disease, a high risk of SCD, and ventricular tachyarrhythmias [33]. ARVC is characterized by a gradual degeneration of RV myocardium, accompanied by the replacement of healthy tissue with fibro-fatty deposits.

Lesions may also manifest in LV myocardium, and predominant LV disease can concurrently exist within the same family. The acknowledgment of LV involvement in many cases of ARVC has led to a focus shift from severe RV disease (so-called “classical ARVC”) to a broader range of different phenotypes with left-dominant or biventricular involvement [34,35,36]. The ESC 2023 guidelines encompass this left-dominant phenotype into the broader group of NDLVC. The NDLVC phenotype includes individuals who have previously been described with varying terms such as arrhythmogenic left ventricular cardiomyopathy (ALVC), left-dominant ARVC, or arrhythmogenic dilated cardiomyopathy (ADCM) (often without meeting the diagnostic criteria for ARVC).

A systematic approach aiming to exclude phenocopies should be undertaken [6].

The differential diagnosis of ARVC includes myocarditis, sarcoidosis, RV infarction, DCM, Chagas disease, pulmonary hypertension, and CHD with volume overload (Ebstein anomaly, atrial septal defect, partial anomalous venous return with a left-to-right shunt).

### 3.2. Epidemiology

ARVC diagnosis ought to be considered in adolescents or young adults who present symptoms of palpitations, syncope, or aborted sudden death (ASD) [6].

The arrhythmic presentation may exhibit variations based on their age. While adult patients tend to frequently experience sustained ventricular tachycardia (VT), children and adolescents are more prone to cardiac arrest (CA) and SCD, which may represent the initial clinical presentation of the disease [37].

Adult prevalence is estimated to be 1:5000 and, even if the specific pediatric prevalence is still not known, it is considered very rare in infants. Whilst age-related penetrance is evident in ARVC, with the highest incidence occurring between the ages of 30 and 40 years, it is noteworthy that no comprehensive studies have been conducted to systematically assess ARVC epidemiology during childhood. Furthermore, the representation of pediatric populations in the medical literature is inadequate, which raises the suspicion of a potential bias toward the underdiagnosis of ARVC in this age group. In 1988, a study conducted on the incidence of SCD among juveniles in the Veneto region of Italy revealed that 20% of SCD cases in this population were attributed to an undetected ACM [38].

These data are confirmed by a more recent paper reporting that 25% of SCD in children and adolescents may be ascribed to ARVC [39].

The first attempt to compare young vs. adult patients with ARVC was performed by Daliento et al. in 1995. Young patients displayed a greater amount of fibrosis on endomyocardial biopsy (EMB) and had more frequent ventricular fibrillation (VF) than adults [40]. In 2011, Bauce confirmed the high risk of life-threatening VA in children with desmoplakin (DSP) mutations [41].

Children’s inheritance is most commonly autosomal recessive (AR), and it is frequently associated with cutaneous manifestations (e.g., Naxos disease and Carvajal syndrome) [42,43]. However, it is important to note that this observation may be indicative of a systematic clinical screening for these conditions during early childhood.

Recent data indicate that approximately 15% of patients diagnosed with ARVC exhibit symptoms during childhood. Furthermore, it has been observed that pediatric ARVC patients tend to present a more severe phenotype and are at a higher risk of SCD [34].

Moreover, there is a growing recognition of acute myocarditis onset of ARVC in children [42,43,44].

The genetic basis of ARVC primarily involves genes encoding for cardiac desmosomes. These proteins include plakophilin-2 (PKP2), desmoplakin (DSP), desmoglein-2 (DSG2), desmocollin-2 (DSC2), and plakoglobin (JUP) [45,46,47,48]. Additionally, pathogenic, or likely pathogenic variants have also been observed in other genes, such as DES [49], TMEM43, and PLN [48]; it is possible to identify these variants in up to 60% of patients diagnosed with ARVC [50].

Clinical presentation and arrhythmic risk stratification are strictly connected to genes involved both in adults and children. For example, the presence of DSP variants is more frequently associated with “hot phase” myocarditis presentation and life-threatening arrhythmias at the onset, unlike PKP2 variants [51,52,53]. The ARVC pediatric population often experiences recurrences of myocarditis-like episodes with more frequent chest pain, higher levels of troponin I/CPK, and greater edema/hyperemia at cardiac magnetic resonance (CMR) compared to adult patients [25,44,51]. The inflammatory theory and the identification of serum pro-inflammatory cytokines have prompted efforts to target the inflammatory pathways as a novel medical therapy. The discovery of serum anti-DSG2, anti-heart, and anti-intercalated disk autoantibodies has also supported an autoimmunity hypothesis, but larger cohorts and prospective studies are needed to confirm pro-inflammatory theory [51,54].

### 3.3. Diagnosis

For more than 10 years, ARVC diagnosis has been based on revised task force criteria published by Marcus et al. in 2010, both in children and adults [55].

In recent times, the Padua criteria have presented a revised version that incorporates LV involvement, although their complete external validation is still pending [56].

These criteria focus on the role of late gadolinium enhancement (LGE) at CMR imaging as a major criterion. The fundamental role and accuracy of CMR for the diagnosis of ARVC have been validated by several studies and confirmed by recent ESC guidelines in children and adolescents [6,25,45,57].

Moreover, the Padua criteria emphasize the assessment of RV dilation/dysfunction, regardless of severity, and isolated regional abnormalities of LV wall motility, which are considered minor criteria. These changes aim to better reflect the segmental nature of fibro-adipose substitution. Furthermore, the significance of isolated premature ventricular contractions (PVCs) has been expanded beyond the absolute number (>500/24 h) to also include considerations about morphology. Additionally, the distinction between major and minor criteria for fibrous replacement at EMB has been eliminated and is now being classified as a major criterion. The detection of epsilon waves on ECG is considered a minor criterion, while late potentials on signal-averaged ECG are no longer included due to their low diagnostic accuracy.

The essential components of the diagnostic evaluation encompass ECG, Holter monitoring, cardiac imaging, genetic testing, and, in specific situations, EMB [6,26,55].

It should be highlighted that there have been concerns regarding the effectiveness of the 2010 task force criteria in diagnosing ARVC in the pediatric population, probably because T-wave inversion in the anterior precordial leads is a common occurrence in pre-pubertal children [58,59]. In this regard, various international guidelines have advised against clinical screening for ARVC in first-degree relatives until they reach the age of 10 to 12 years [26,60].

In this context, the paper recently published by Smedsrud et al. plays an important role in the knowledge of childhood-onset ARVC. They report findings from a single national referral center of a group of 62 children (under 18 of age), including both individuals affected by a familial pathogenic variant and carriers of the heterozygous phenotype negative. ARVC defined by the 2010 task force criteria (TFC) has been diagnosed in 32% (including 11 probands). It is worth noting that a significant proportion (40%) of children were diagnosed with ARVC before the age of 12. Furthermore, 23% of the entire cohort experienced serious cardiac events, which included arrhythmic events as well as events requiring heart transplantation or related to heart failure (HF). Of note, half of these events occurred in children under the age of 12 [34].

Deshpande et al. proposed the need for a revision of the 2010 International Task Force (ITF) criteria, particularly for pediatric patients, as there is a potential underestimation of the occurrence of ACMs [61]. These data were confirmed by a more recent work at Bambino Gesù Pediatric Hospital. Cicenia et al. assessed the concordance between the 2010 ITF criteria and the 2020 Padua in a pediatric cohort of 21 pediatric patients affected by ACMs [62]. The Padua criteria resulted in more accuracy in the pediatric setting compared to the 2010 ITF criteria, avoiding diagnosis underestimation. Moreover, most patients presented PVCs, stable VA, and fibrous myocardial substitution. Noteworthy, a significant proportion of these children exhibit PVCs with a left bundle branch block (LBBB) morphology and inferior axis, originating from the right ventricular outflow tract (RVOT). This observation may imply that in pediatric patients, this morphology warrants the same level of consideration as the non-RVOT morphology, which is typically given greater attention in adult patients.

In fact, as highlighted by Hoffmayer et al. and Novak et al. in adult populations, the differential diagnosis between idiopathic RVOT arrhythmias and early ARVC is not so well defined and can be extremely challenging. QRS morphological features and duration, QRS notching, and coupling the interval of ventricular ectopic beats (VEBs) should be considered [63,64].

Furthermore, the behavior of VA during exercise testing does not seem to help find definitive conclusions in cases of suspected ARVC, both in Cicenia et al.’s and Sequeira et al.’s studies. Specifically, these papers highlight the importance of not automatically dismissing “benign PVCs” if they disappear during exercise [65,66].

### 3.4. Risk Stratification

It is noteworthy that the progression of structural ventricular alterations can be preceded and predicted by ECG depolarization abnormalities [67].

ECG abnormalities, such as T-wave inversion in right precordial leads or other leads, delayed S-wave upstroke in right precordial leads, right bundle branch block (RBBB), and low voltages in limb leads, along with VA, including isolated PVCs and non-sustained or sustained VT, serve as manifestations of these histologic changes and may occur prior to the development of structural phenotypic alterations [33,68].

Moreover, in the adult population, pathological Q waves, a left posterior bundle branch block, and a prominent R-wave in V1 are recently described as common ECG signs of arrhythmogenic left ventricular cardiomyopathy (ALVC) [69].

Patients with more than one gene mutation were found to have a worse outcome and a higher chance of arrhythmias; specific genes, like DSP and PLN, increase the risk of LV disfunction and HF [70]. For instance, DSP LP/P variations and PLN-pArg14 have been linked to a worse prognosis and increased risk of SCD. In addition, FLNC and DES mutation carriers have a significant risk of SCD due to a ring-like pattern of subepicardial LV fibrosis [71,72].

In 2015, Corrado et al. proposed an algorithm for risk stratification of arrhythmic patients based on the consideration of “major” and “minor” risk factors. This algorithm categorizes patients into three different levels of arrhythmic risk: high, moderate, and low. The major risk factors considered for risk stratification include cardiac arrest or life-threatening arrhythmias, non-sustained VT (NSVT), the degree of heart dysfunction (moderate or severe dysfunction in the RV or LV), and syncopal events. On the other hand, the minor risk factors encompass proband status, the male gender, electrical instability (spontaneous VA or arrhythmias induced during electrophysiological (EP) study), a younger age, complex genotypes, and the extent of SHD [73,74]. Furthermore, two additional algorithms have been proposed more recently for the prediction of life-threatening VA [75,76].

Several recent expert consensus documents have synthesized the available evidence on arrhythmic risk stratification in ARVC. These include the 2015 ITF consensus statement on the management of ARVC, the 2017 American Heart Association/American College of Cardiology/Heart Rhythm Society guideline for the management of VA, and the 2019 Heart Rhythm Society consensus statement on the evaluation, risk stratification, and management of ACM [26,74,77].

While these publications have significantly advanced the knowledge and guidance for clinicians caring for ARVC patients, they still have certain limitations. Firstly, these algorithms were primarily based on expert opinion rather than robust empirical evidence. Secondly, all the guidelines were presented in a flowchart format, which did not account for the potential interactive effects of combinations of risk factors. Lastly, the translation of these guidelines into absolute risks was not adequately addressed [78].

Cadrin-Tourigny et al. in 2019 created a prediction model of life-threatening VA and SCD in ACM patients to overcome some of these studies’ limitations [76].

The authors proposed an algorithm that aims to assess the risk of SCD as a continuous variable. This algorithm utilized six well-established risk predictor variables: sex, age, recent cardiac syncope within the past six months, NSVT, number of PVCs detected on 24 h Holter monitoring, extent of T-wave inversion on anterior and inferior leads, RV ejection fraction (EF), and LV EF. The primary outcome was the occurrence of the first sustained VA that was defined as sustained VT, the presence of SCD, VF/flutter, or appropriate implantable cardioverter defibrillator (ICD) intervention. This model allowed for appropriate patient selection, avoiding unnecessary ICD implants. Specifically, the implementation of this model resulted in a 20.6% reduction in the number of ICD implantations compared to the current algorithm while providing a greater overall benefit in terms of protection. It is important to note that this study, although the largest conducted prior to 2019 with a sample size of 528 patients, had some limitations: a higher prevalence of Caucasian patients, a higher prevalence of PKP2 variants identified, and the use of ICD shocks as a surrogate marker for SCD. This surrogate measure was employed to lower the risk of overestimating VA cases. This approach was supported by the understanding that stable VAs, even if sustained, do not share the same predictive factors as fatal VAs. The Cadrin–Tourigny risk score has demonstrated validity in cases of isolated RV presentation. However, its applicability in LV involvement is still not clear [72,73].

Furthermore, it is crucial to emphasize that only a minority of the study participants are in the pediatric age range. Therefore, it is necessary for future research to validate the effectiveness of the risk model in children and adolescents.

The risk of disease at any age is highly variable and heterogeneous and may be caused by multiple underlying factors. Therefore, the risk should be tailored to the individual patient, and the most up-to-date information should be considered in conjunction with older guidelines. The absolute risk may vary over time in individual cases, and a periodic reassessment in pediatric age should be warranted.

### 3.5. Treatment

Exercise is a known risk factor in patients with ARVC. Several studies have shown that endurance training is associated with increased disease progression, increased risk of arrhythmias, and adverse cardiovascular outcomes in ARVC patients [79,80]. There has been limited information specifically focused on the impact of exercise on pediatric patients. The cornerstone of pharmacotherapy for ARVC is the use of beta-blockers. Angiotensin-converting enzyme (ACE) inhibitors play an important role in the treatment of significant structural disease and RV and/or LV dysfunction. Radiofrequency ablation with a primary epicardial or combined endo-epicardial approach can be useful in patients with high VT recurrence [81,82]. SCD prevention with ICD placement is of primary importance. However, this treatment is invasive, carries risks of complications, and can cause physical or psychological distress to patients. Estimating the likelihood of developing ventricular arrhythmias and developing a risk score is, therefore, important to protect those at high risk while limiting interventions to those who are unlikely to benefit from the outcome. However, for the purpose of this review, it should be emphasized that the number of study participants in the pediatric age group was usually small, and future studies should confirm the utility of risk models in children and adolescents.

## 4. Dilatated Cardiomyopathy (DCM)

Systolic dysfunction and LV dilation that cannot be fully explained by abnormal loading circumstances or coronary artery disease are the hallmarks of DCM. LV dilation is defined when end-diastolic dimensions or volumes exceed population mean values after adjusting for age, gender, and/or body size. In adults, LV dilatation is defined by an LVEDV index of 62 mL/m^2^ in females and 75 mL/m^2^ in males (corresponding to an LV end-diastolic diameter >58 mm in males, and >52 in females), and in children by z-score > 2 DS [6].

### Epidemiology and Genetics

Children typically experience an incidence of 0.57 per 100,000, with boys 0.66 and girls 0.47 per 100,000 [83,84]. Idiopathic forms account for about 66% of cases. Sometimes the disease is incidentally found and frequently underdiagnosed [78,79].

When DCM is diagnosed in children, reversible causes, such as hypocalcaemic vitamin D-dependent rickets or viral myocarditis, should be ruled out [85,86,87]. Moreover, CHD-like aortic coarctation or an anomalous origin of the left coronary artery (ALCAPA) should be excluded [84,88]. In children with hypotonia and increased creatine kinase, it is necessary to rule out dystrophin- and sarcoglycan-related CM with a multidisciplinary approach involving a neurologist and experts in metabolic disease [82,83]. Barth syndrome and other mitochondrial/metabolic disorders should be taken into consideration when a DCM phenotype is linked to LV hypertrabeculation.

The DCM that develops in pediatric patients is a distinct clinical entity from adult disease: it is characterized by the worst prognosis and carries more HF and arrhythmic events. This results in the need for more aggressive therapies and follow-up in pediatric DCMs, implying challenging clinical related to patients’ young ages [19,89,90]. It is largely unknown why children exhibit this particular aggressive form.

In the clinical assessment of pediatric-onset CM, genetic testing plays a significant role. Finding a variant in a related gene can be used to confirm the clinical diagnosis and rule out syndromic causes that may call for different treatment approaches.

The development of DCM is linked to over 110 genes, including sarcomere genes (MYH7, TPM1, and TTN), calcium-sensitive and calcium-signaling genes (JPH2, and PLN), z-disk genes (ACTN2, BAG3, and CRYAB), and nuclear envelope genes (LMNA, EMD) [91].

Up to 25% of familial cases of DCM are associated with truncating titin variants, or TTNtvs, which are by far the most prevalent genetic cause of the disease [92].

A prognostic guide to treatment options can be provided by genotype–phenotype correlations that have been established in pediatric DCM patients. For instance, some TTNtvs, which by themselves are not linked to the emergence of DCM, raise one’s vulnerability to stress-induced CM, such as those brought on by pregnancy or doxorubicin chemotherapy [93,94]. As a result, it is imperative that TTN variant carriers in these situations—especially those who have affected family members who also carry the variant—be closely followed. Typically, DCM is inherited in an AD manner; however, AR inheritance has also been documented, for instance in JPH2 [95]. The Lamin A/C (LMNA) variant is often shared with ACM and encompasses an arrhythmic DCM phenotype [96,97,98]. RBM20, PLN, and FLNC are additional genes linked to arrhythmic DCM phenotypes [99,100,101,102,103].

There is genetic overlap between DCM, HCM, and the other CMs, indicating that all CMs share some phenotypic characteristics. In a cohort of 639 patients with familial or sporadic DCM, for instance, 31% of identified variants were also linked to ACM and 16% to HCM [104]. The yield of genetic testing in familial DCM in clinical practice ranges from 30% to 40% [105,106].

The need for EP evaluation of at-risk individuals and possible ICD placement can be guided by the identification of genes with arrhythmic potential.

In a large cohort of 285 adult patients with DCM extensively investigated and followed for >20 years, the prevalence of the AR-DCM phenotype was approximately one-third of the overall DCM population. Unrelated to the degree of LV dysfunction, VA may first appear early in the course of the disease [107].

A wide variety of bradyrhythmias and tachyarrhythmias, including sinus node dysfunction, different degrees of atrioventricular block (AV block), interventricular conduction delay, and atrial and VA, can occur in patients with DCM. Six percent of DCM patients have LMNA mutations, and 33% of DCMs with AV block have LMNA mutations [108,109]. Conduction abnormalities can develop years before HF or LV dysfunction, so the onset of AV conduction defects in middle age or earlier should prompt an evaluation for inflammatory or familial cardiomyopathy. Even in the presence of normal LV function, a close follow-up is required. An “irritable focus” caused by myocardial fibrosis, elevated catecholamine levels, or stretching of the myocardial fibers can be used to explain the arrhythmogenic substrate. The ion channel function may also change because of the breakage of the connection between the sarcolemma, cytoskeleton, and sarcomere. Patients may exhibit overt HF symptoms, such as dyspnea, sweating, and reduced exercise tolerance. Cachexia and decreased appetite are common in young children. Clinical signs include hepatomegaly, pallor, sinus tachycardia, and distension of the jugular venous pressure (JVP) [19].

Atrial ectopic tachycardia and permanent junctional reciprocating tachycardia (PJRT) are the two supraventricular arrhythmias that most commonly cause arrhythmia-induced cardiomyopathy (AIC) in the pediatric population. VA is infrequently found to be the root of HF in children [110]. Even though growing evidence points to some inherent differences in myocardial adaptations brought on by DCM, the pathophysiology of HF in children is similar to adults. The natural course of pediatric DCM differs from adult DCM in complications’ time of onset. Death or transplantation typically takes place within 2 years of DCM presentation, indicating that many children and adolescents already have advanced disease at the onset [84,111].

Differences between adults and children may also be explained by different cardiac receptor behavior. In patients with idiopathic DCM, both children and adults have decreased expression of the total myocardial β-adrenergic receptor; however, in adults, only the β-1 adrenergic receptor is downregulated, while in children, both the β-1 and the β-2 adrenergic receptors are downregulated. The varying reactions observed in clinical trials among children with HF receiving adult-developed therapies could be attributed to these distinctions [112]. The effects of the medical blockade of the already downregulated β-2 adrenergic receptors are unknown, and this differential receptor expression may affect how children respond to nonselective β-blockers, like carvedilol [113].

The stratification of mortality, transplant, and recovery still requires a unified clinical risk algorithm in children. In the PCMR (Pediatric Cardiomyopathy Registry) of 1803 children with DCM, the 5-year incidence rates were 29% for heart transplantation, 12.1% for non-SCD, 4.0% for death by an unknown cause, and 2.4% for SCD. The risk stratification model included age at diagnosis younger than 14.3 years, LV dilation, and LV posterior wall thinning [114]. Even though the 1- and 5-year survival rates have steadily increased, more children with DCM are receiving cardiac transplants, and the event-free survival rate remains roughly the same as it was decades ago. Multicenter studies are needed to identify SCD risk stratification, pre-transplant prognostic factors, and post-transplant survival experience.

## 5. Hypertrophic Cardiomyopathy (HCM)

### 5.1. Introduction and History

HCM is defined as the presence of increased LV wall thickness (with or without RV hypertrophy) or mass that is not solely explained by abnormal loading conditions [115].

The first contemporary morphological description of HCM was reported in 1958 by Teare [116], who reported a case series of eight patients aged 14–44 years, seven of whom succumbed to sudden death (SD), with pathological findings of asymmetric ventricular hypertrophy characterized by an irregular arrangement of myocardial muscle bundles and the presence of fibrotic tissue.

Among the pediatric population, HCM is the second most common CM [117], representing a heterogeneous condition in terms of etiology, clinical presentation, genomic alterations, and survival outcomes.

Although there is a large body of literature about HCM in adults, a significant research gap still exists for the pediatric population.

### 5.2. Epidemiology

Studies regarding the epidemiology of the condition are mostly limited to North America and Europe, which have investigated the annual incidence of HCM in children [16,17,18,118]. The Nationwide Study in Finland carried out the first well-defined population-based retrospective study to determine the epidemiology of idiopathic CMs in children, including HCM [118]. During the 12-year study period, the estimated annual incidence of HCM in children was 0.24 per 100,000 children under 20 years of age. In 2003, the National Australian Childhood Cardiomyopathy Study and the Pediatric Cardiomyopathy Registry of the United States presented an annual incidence of HCM in children of 0.32 per 100,000 children below 10 years of age and 0.47 per 100,000 children below 18 years of age, respectively [16,17].

In China, based on the studies available at present, HCM does not appear to be rare. In fact, based on the estimated prevalence, there are at least 1 million cases in China [119].

Therefore, according to the latest ESC guidelines for the management of CMs and the current state of the art, the estimated overall prevalence and incidence would seem to be 0.002–0.005% and 0.029%, respectively [6].

### 5.3. Genetics

The disease is believed to be hereditary in 90% of instances, typically following an AD inheritance pattern, except for situations involving alterations in mitochondrial (MT) DNA, which are inherited from the mother [120,121].

Alterations have been documented in various genes that encode vital sarcomeric proteins, including heavy chain b-myosin (MYH7), myosin-binding protein C (MYBPC3), heavy chain a-myosin (MYH6), troponin I (TNNI3), troponin T (TNNT2), a-tropomyosin (TPM1), essential myosin light chains (MYL3), regulatory light chains (MYL2), titin (TTN), and α-actin (ACTC) [122].

The most frequently affected genes are the myosin-binding protein C and the β myosin heavy chain, accounting for over 70% of genotyped patients. Sporadic cases due to de novo genetic mutations have also been reported [122]. The phenotypic expression of HCM may first occur at all phases of life, from infancy to old age.

Mutations have also been observed in genes linked to heme and Fe^2+^ group metabolism, as well as genes involved in MT bioenergetics [123].

Indeed, some studies showed evidence of global energetic decompensation manifested by a decrease in ATP, ADP, and phosphocreatine associated with mutations in MT genes involved in creatine kinase and ATP synthesis. Furthermore, electron microscopy showed an increased fraction of severely damaged mitochondria with reduced cristae density, coinciding with reduced citrate synthase activity and MT oxidative respiration. These MT abnormalities were associated with elevated reactive oxygen species and reduced antioxidant defenses. These results highlight potential new drug targets for attenuation of the clinical manifestations by improving metabolic function and reducing MT injury [18].

Genetic investigations of families with LV hypertrophy revealed metabolic CMs caused by mutations in the PRKAG2 and LAMP2 genes.

Mutations in the PRKAG2 gene, which encode the γ_2_ subunit of the adenosine monophosphate-activated protein kinase, cause Anderson–Fabry disease, the most common metabolic disorder in adults with HCM [124]. Instead, mutations in the lysosome-associated membrane protein 2 (LAMP-2) gene cause Danon disease, which is typically fatal by young adulthood [125].

Although still rare, metabolic disorders account for a greater proportion of HCM in children and adolescents.

Syndromes associated with HCM include Noonan syndrome (and other RASopathy syndromes), which may present in infancy with biventricular hypertrophy, and Freidrich’s ataxia, which has progressive hypertrophy throughout childhood with decreasing systolic function in adolescence [122].

Recently, missense mutations leading to impaired interaction between nexilin (a cardiac Z-disc protein crucial for safeguarding cardiac Z-discs against internal sarcomere-generated forces) and α-actin have been identified in HCM [126].

So far, mutations have not been considered predictive of phenotype severity because individuals from the same family carrying the same mutation may exhibit varying degrees of hypertrophy or a differing predisposition to SCD [1,127,128]. This is due to the influence of modifier genes and polymorphisms, necessitating more comprehensive research. It is hypothesized that the disruption of MT energy metabolism in the heart is the underlying cause of HCM in cases of sarcomeric contraction disturbance. This insight sheds light on various clinical observations, including heterogeneity, variability in clinical presentation, and irregularities in hypertrophy [1].

### 5.4. Clinical Presentation

HCM may present in children with different clinical manifestations. Patients may be asymptomatic or may seek medical attention for findings of clinical HF or an abnormal ECG, may have a member of their family with HCM, or may present an aborted sudden cardiac arrest or SCD.

In infants, some patients experience symptoms and signs of HF, including tachypnoea, poor feeding, excessive sweating, and failure to thrive. Older children, adolescents, and adults complain of fatigue and dyspnoea, as well as chest pain, palpitations, and syncope [129].

Several non-cardiac symptoms act as pointers for specific diagnoses such as learning difficulties and palpebral ptosis in Noonan/LEOPARD syndrome, gait disturbance in Friedreich’s ataxia, angiokeratoma, hypohidrosis, and cataracts in Anderson–Fabry disease [129]. Similarly, general physical examination can provide diagnostic clues in patients with syndromic or metabolic causes of HCM. Paradoxically, the cardiovascular examination is often normal but in patients with LV outflow tract obstruction (LVOTO), a few typical features may be identified, such as a rapid up-and-down stroke to the arterial pulse and a loud crescendo–decrescendo ejection systolic murmur, which is located along the left sternal border and radiates to the neck; the murmur is typically louder with maneuvers that decrease the preload of the LV, such as standing and Valsalva maneuver. More ominous modes of presentation, associated with a greater risk for poor outcomes, include clinical outset with HF symptoms, syncope, arrhythmias, and aborted SCD [130].

Additionally, progressive LV diastolic dysfunction can lead to progressive left atrial (LA) enlargement that can predispose HCM patients to develop atrial arrhythmias and pulmonary hypertension. Older children with HCM may experience progressive LV dysfunction and dilation with a transition to dilated phenotype and chronic systolic HF over time. This phase of the disease is called “burned-out HCM” and refers to the end-stage of HCM, characterized by myocardial fibrosis, systolic dysfunction, and LV wall thinning [131]. Reversible myocardial ischemia and perfusion defects occur commonly in HCM, especially during exercise or higher heart rates, leading to the symptoms of chest pain, dyspnea, lightheadedness, or syncope [132].

### 5.5. Electrocardiographic Findings

ECG findings in adult patients with HCM have been extensively documented and include increased voltages, pathologic Q waves, ST-segment depression, T-wave inversion, and axis deviation. Research has shown that approximately 90% of adult HCM patients exhibit abnormal ECG patterns [133,134].

However, large-scale descriptive studies focusing on the pediatric HCM population are lacking. Consequently, the incidence and patterns of abnormal ECGs in children with HCM are often extrapolated from the adult literature [135]. A comprehensive understanding of ECG abnormalities observed in pediatric HCM is crucial for the early identification of at-risk patients.

ECG findings in HCM patients are known to precede echocardiographic findings [133]. Early diagnosis via ECG may prevent children and adolescents from competitive sports-related SCD or aborted CA.

In a multicenter international study conducted by the Pediatric and Congenital Electrophysiology Society (PACES) group on HCM, ECG data were collected from children and adolescents aged 21 or younger with “idiopathic” or “sarcomeric” HCM (patients with genetic syndromes, such as Noonan’s syndrome, storage disorders, like Pompe’s disease, and neuromuscular disorders, such as Friedrich’s ataxia, were excluded). This study found that 88% of patients exhibited ECG abnormalities [136].

The three primary patterns observed in those with abnormal ECGs included left ventricular hypertrophy (LVH) or biventricular hypertrophy, using the criteria of Saarel et al. [134] for the diagnosis of hypertrophy, with or without strain, and pathologic Q waves in the inferior leads. Nine percent of patients exhibited features of ventricular strain patterns without associated hypertrophy. Isolated LVH by voltage criteria is a common finding in children. Previous population-based ECG studies [137,138] have indicated that the prevalence of this finding in otherwise healthy children can vary and depends on the specific criteria used. Several pediatric-specific criteria have been proposed to enhance accuracy in identifying children with HCM. Studies have revealed a poor correlation between precordial voltage on ECG and echocardiographic LV mass. One study demonstrated [139] that 50% of normal adolescent athletes met voltage criteria for LVH. Nevertheless, pathological Q waves, T-wave inversion, ST-segment depression, and the presence of more than one ECG abnormality were useful in distinguishing HCM features from those of healthy athletes [136].

Pathologic Q waves, a finding often emphasized in the adult literature, are less frequently emphasized as a finding in pediatric HCM. Previously, various definitions were used to differentiate pathologic Q waves from those in healthy athletes. Konno et al. conducted a study to define pathological Q waves. In a cohort of HCM patients confirmed by genetic diagnosis, they established that Q waves exceeding 3 mm in depth and/or lasting longer than 0.04 s in at least two leads (except aVR) were the most accurate (69%), sensitive (50%), and specific (90%) criteria for diagnosing Q waves in individuals under 30 years of age [140]. Similarly, Charron et al. studied a pediatric cohort with known genotypic mutations for HCM and found abnormal Q waves to be a major diagnostic criterion with 100% specificity [141]. Bent et al. demonstrated that Q waves exceeding 30 ms in lead I had the greatest discriminatory value between athletes and HCM patients [142]. Sato et al. showed that abnormal Q waves reflected the area of hypertrophy, with Q waves in I and aVL reflecting hypertrophy of the septum, posterior/lateral wall base, and apex. T-wave morphology was associated with hypertrophy in basal versus apical segments rather than absolute muscle thickness [134].

Therefore, normal ECG or ECG abnormalities that do not exhibit classic ventricular hypertrophy or strain patterns should not rule out the possibility of HCM. If patients present concerning symptoms and exhibit the aforementioned abnormalities on their ECG, further evaluation, including an echocardiogram, should be considered.

### 5.6. Diagnosis

The initial diagnostic evaluation for HCM involves a comprehensive assessment that includes personal and family medical history, physical examination, ECG, cardiac imaging, and initial laboratory tests.

The diagnostic criteria differ from adults to pediatric patients affected by HCM.

In the adult population, HCM is defined by the presence of an LV wall thickness of 15 mm or greater in any myocardial segment, which cannot be solely attributed to loading conditions. When there is lesser wall thickening (13–14 mm), further evaluation is required, considering additional factors such as family history, genetic findings, and abnormalities in the ECG [129].

In children, the diagnosis of HCM requires the presence of LV wall thickness exceeding two standard deviations beyond the predicted mean, denoted as a z-score > 2 [143].

For relatives, in adult first-degree relatives of individuals with confirmed HCM, the clinical diagnosis is established when LV wall thickness is equal to or exceeds 13 mm. In the case of child first-degree relatives, if the LV wall thickness z-scores are less than 2, the presence of associated morphological or ECG abnormalities should raise suspicion, although these findings alone are not diagnostic for an HCM [129].

The construction of a multi-generational family pedigree is a valuable tool for confirming the genetic basis of a disease and identifying family members at risk. It involves noting specific details in the family’s medical history, such as SCD, unexplained HF, cardiac transplants, the presence of pacemaker (PMK) or defibrillator implants, and signs of systemic disorders, like early onset stroke, muscle weakness, kidney problems, diabetes, or hearing issues. A pedigree analysis also helps determine the likely inheritance pattern. Most genetic forms of HCM follow an AD pattern, where affected individuals appear in every generation and can be transmitted by parents of any gender (even from father to son) and carry a 50% risk of transmitting the condition to offspring. In cases where males are severely affected but male-to-male transmission is absent, X-linked inheritance should be considered. AR inheritance is less common and typically occurs when both unaffected parents are closely related. Additionally, when only females transmit the disease to children of both sexes, MT DNA mutations should be considered in the investigation [129].

### 5.7. Treatment

The management of HCM is centered on the mitigation of its clinical manifestations, the optimization of hemodynamics to minimize dynamic LVOTO, and a reduction in SCD risk. Key aspects of HCM patient management are presented below.

1. *Pharmacological Intervention*: Beta-blockers should be considered for neonates and children exhibiting LVOTO. Medical therapy may be contemplated in asymptomatic or mildly symptomatic adolescents and adults with resting or provoked LVOTO and left atrial enlargement. Propranolol [129] can be initiated at 1.5–3 mg/kg/day and uptitrated to a minimum of 6 mg/kg/day, with further adjustments (up to 23 mg/kg/day) based on therapeutic response and heart rate control as assessed via Holter recordings. In cases of bronchospasm or hypoglycemia, metoprolol can be considered at doses of 6–12 mg/kg [144,145]. Limited data also suggest the safe use of verapamil in children. Verapamil should be used cautiously in children with LVOT obstruction due to potential peripheral vasodilation and hemodynamic complications. Paradoxical LVOT gradient increases can occur even with small verapamil doses, necessitating vigilant monitoring and prearranged short-term clinical and hemodynamic assessments in cases of β-blocker ineffectiveness [146].

Disopyramide, with its negative inotropic action, may reduce gradients and relieve symptoms. However, it is best used in conjunction with β-blockers, although specific recommendations for disopyramide therapy in children are lacking [147].

Diuretics are generally discouraged in the early stages of HCM, even with evidence of left atrial hypertension. This approach aims to avoid volume depletion, which can worsen ventricular filling. Diuretics may be judiciously used in later stages when left atrial hypertension and congestive HF symptoms become prominent. Moreover, it should be noted that pediatric HCM patients may be more vulnerable during common childhood illnesses, like gastroenteritis or bronchiolitis, as these can lead to increased volume losses, with subsequent worsening of symptoms.

2. *Exercise Avoidance*: Patients with HCM are advised to abstain from high-intensity exercise, as it can exacerbate symptoms, particularly dynamic obstruction, and are encouraged to perform controlled moderate-intensity exercise [148].

This is a very sensitive aspect of the issue because young patients typically appear in excellent health, do not experience symptoms, and often show interest in engaging in a vigorous lifestyle, including various sports. Therefore, it is necessary to explain the problem adequately, perhaps with the assistance of an expert, as this can also have psychological implications for the child’s health.

3. *Surgical intervention*: Patients with severe symptoms, large LVOT gradients, and poor response or intolerance to medical therapy may require more invasive interventions.

*Myectomy*: Myectomy is traditionally regarded as the preferred treatment for patients with drug-resistant symptoms and a resting gradient of ≥50 mmHg. It typically yields symptomatic improvement in about 70% of patients for at least 5 years. Possible complications include ventricular septal defects, third-degree AV block necessitating PMK implantation, cerebral embolism, and postoperative LBBB. About 10% of pediatric patients may require a repeat myectomy due to LVOT obstruction recurrence [144,149].

*Alcohol-Induced Septal Ablation*: Alcohol-induced septal branch occlusion has been applied to younger patients and has been recognized its effectiveness and low complication rates. Indications for percutaneous septal ablation include symptomatic patients with functional class III symptoms despite optimal drug therapy, substantial medication side effects, high resting or stress gradients (≥50 mmHg at rest or ≥100 mmHg under stress), and documented risk factors for SCD and/or reduced exercise capacity. However, it should be noted that gradient reduction does not necessarily correlate with reduced SCD risk [150].

Several technical considerations are worth noting, especially when dealing with children and adolescents. The size of their coronary arteries may pose a limitation since the guide catheter typically requires at least a 6F width, and in some cases, 7F is necessary for adequate support. Additionally, the commercially available balloons may not always be proportionate to the smaller septal branches in pediatric patients. It is crucial to emphasize these aspects to prevent any harm to the coronary arteries. Furthermore, in younger patients, it appears that the ventricular septum is well-perfused, likely due to the presence of collateralized vessels. This may result in a quicker dispersal of the injected alcohol during percutaneous septal ablation (PSA). Overall, the outcomes of PSA are highly satisfactory, with a substantial reduction in the LVOT gradient observed in approximately 90% of patients in the short term [150]. Mortality rates during this procedure range from 0% to 4%. The most common complication is the AV conduction block, which necessitates permanent PMK implantation in less than 10% of treated patients. It is important to note that these complications, along with issues related to access sites, have not been observed in pediatric patients.

Additionally, the early and intermediate results of PSA are comparable to those following myectomy [151]; however, it should be noted that younger patients tend to experience a smaller reduction in LVOT gradient after the procedure [152]. This phenomenon may be attributed to the substantial collateralization of the septum, as previously mentioned. Furthermore, it is likely that young patients exhibit a higher degree of fibrosis, as opposed to muscle tissue hypertrophy, in the frequently thickened septum. This fibrosis may lead to inadequate scar formation following PSA. In the future, advances in tissue characterization may offer solutions by enabling the distinction between muscle and fibrotic tissue, thus facilitating more efficient pre-interventional assessments.

4. *Heart Transplantation*: For patients with progressive HF unresponsive to medical treatment, heart transplantation remains the ultimate therapeutic option.

In summary, the management of HCM is multifaceted and individualized, aiming to alleviate symptoms, optimize cardiac function, and minimize associated risks. Treatment strategies are selected based on disease stage, severity, and patient-specific factors.

### 5.8. Prevention of SCD

SCD is the predominant cause of mortality during childhood and adolescence [117,153,154], and one of the pivotal aspects of clinical management in pediatric HCM is the identification of individuals with the highest SCD risk. Initial studies conducted on small, highly specific cohorts of children suggested an annual SCD incidence of up to 7% [155,156]. However, more recent data from larger population-based studies have revealed SCD rates ranging between 0.8% and 2% per year [117]. These rates are notably lower than the earlier reports but are still substantially elevated compared to those observed in adults with HCM.

The primary strategy for preventing SCD involves the use of ICDs, which have demonstrated effectiveness in terminating malignant VA in both pediatric and adult HCM populations. Nevertheless, ICD implantation can be associated with significant morbidity. Hence, it is imperative to accurately identify those children at the highest risk who would derive the greatest benefit from ICD placement while minimizing the potential for complications.

There are some risk factors with good evidence of an association with SCD risk in childhood with HCM such as unexplained syncope, LVH, NSVT, and LA enlargement. Other additional risk factors with less conclusive or emerging evidence of an association with SCD in HCM are LVOTO, LGE on CMR, restrictive physiology at the echocardiogram, ventricular and atrial myocardial strain, and exercise-induced ischemia [148].

The etiological factors underlying the disease appear to exert an influence on the risk of SCD, although they do not function as independent risk factors for SCD in children with HCM. In the North American Pediatric Cardiomyopathy Registry cohort, the incidence of SCD was notably higher in patients with non-syndromic HCM compared to those with malformation syndromes or inborn errors of metabolism [157]. This observation was further substantiated by Norrish et al. [153], who examined a cohort of 687 pediatric HCM patients (ranging from 0 to 16 years of age) and found that those diagnosed in infancy or presenting inborn errors of metabolism had a more unfavorable prognosis. Interestingly, no arrhythmic events occurred in 78 children with Friedreich’s ataxia-related HCM, suggesting that these patients may be at a lower risk of VA. However, further studies are necessary to confirm the results observed in this population of patients with Friedreich’s ataxia and to elucidate whether there are any mechanisms that can explain this reduced predisposition to develop VA.

Among the RASopathies, patients with Noonan syndrome with multiple lentigines (previously known as LEOPARD syndrome) appear to carry a higher risk.

The 2014 guidelines from the ESC introduced a risk calculation algorithm called HCM Risk-SCD for adult patients with HCM. However, this algorithm was not validated for patients under the age of 16. In the guidelines, a brief section addresses risk stratification in children and acknowledges the challenge of limited data. It suggests that certain factors, such as unexplained syncope, severe LVH, NSVT, and a family history of SCD, could be considered major risk factors. It also proposes that having at least two of these risk factors could indicate the consideration of implanting a primary prevention ICD. Nevertheless, a formal evaluation conducted by Norrish et al. on a cohort of 411 patients in the United Kingdom revealed that this approach, when applied to children, lacks sufficient sensitivity for practical clinical use [158].

The updated American Heart Association/American College of Cardiology (AHA/ACC) guidelines, published in 2020 [159], include brief sections regarding recommendations for pediatric patients, which are quite similar to those from 2011. They recommend the implantation of a primary prevention ICD in children who have one or more of the following risk factors: unexplained syncope, significant LVH, NSVT, or a family history of SCD.

However, these guidelines acknowledge that the positive predictive value of this approach is not very reliable, especially in children. As a result, there is a recognition of the need for new risk factor algorithms that can offer higher positive predictive value, particularly in the pediatric population.

After recognizing the limitations of the 2014 pediatric guidelines for HCM [129], a significant effort was made to gather a large group of pediatric HCM patients to create a specific risk assessment algorithm. This effort involved collecting data from 1024 patients across 39 participating centers [160]. The newly developed risk score, called HCM Risk-Kids, included LV wall thickness (expressed as a Z-score), LA diameter Z-score, unexplained syncope, NSVT, and LVOTO gradient. In 2022, a comprehensive external validation study was conducted, demonstrating that HCM Risk-Kids, with a risk threshold of >6% at 5 years, could identify over 70% of children at risk of a major arrhythmic event, with a C-statistic of 0.75 [154]. Additional validation from a national cohort showed similar performance, with a C-statistic of 0.69, a sensitivity of 73%, a positive predictive value of 22%, and a negative predictive value of 95%. Furthermore, the study found that incorporating data from patients diagnosed in infancy and combining HCM Risk-Kids with an ECG risk score improved the specificity and C-statistic compared to using either measure alone [161].

After the introduction of the HCM Risk-Kids model, a separate pediatric-specific model was developed in a North American multicenter retrospective cohort (PRIMaCY) comprising 572 patients up to 18 years old. This model was validated using data from 285 patients in the SHaRe registry [162]. The risk factors included in this model were selected based on their association with the endpoint of an arrhythmic event and encompassed age at diagnosis, documented NSVT, unexplained syncope, septal diameter z-score, LV posterior wall diameter Z-score, LA diameter z-score, and peak LVOTO gradient. The authors assessed the performance of the model by dividing the cohort into three risk tertiles (<4.7%—low, 4.7–8.3%—medium, and >8.3%—high risk at 5 years), which showed good agreement and had large confidence intervals.

Based on these data, the AEPC Working Group on Basic Science, Genetics and Myocardial Disease and the AEPC Working Group on Cardiac Dysrhythmias and Electrophysiology believe that HCM Risk-Kids outperforms the risk assessment strategies of the ESC 2014 and AHA/ACC 2020 guidelines. However, further independent external validation studies are needed to directly compare the performance of HCM Risk-Kids and the PRIMaCY model in clinical practice. It is also suggested that the inclusion of additional clinical risk factors may enhance their predictive accuracy [148].

When deciding whether primary prevention ICD therapy is necessary in pediatric patients, a careful evaluation of risks and benefits is essential. These factors should be thoroughly examined, balanced, and discussed with pediatric patients and their caregivers, resulting in a shared decision regarding ICD implantation.

Transvenous, non-transvenous, and subcutaneous ICDs are equally effective for primary and secondary prevention of SCD in pediatric HCM [153,157]. Dual-chamber ICDs do not demonstrate superiority over single-chamber ICDs in discriminating supraventricular or sinus tachycardia. Lead failure issues are more prevalent in non-transvenous systems compared to transvenous systems, and the simplest ICD systems are associated with the lowest likelihood of lead-related complications [69,148,163].

## 6. Brugada Syndrome (BRS)

### 6.1. Introduction

BrS is an AD, an inherited channelopathy characterized by a disturbance in the sodium ion current. Discovered three decades ago [164], its pathophysiology and mechanisms have not completely been clarified yet. Its most classic form of presentation includes a typical ECG pattern consisting of ST-segment elevation in precordial leads V1–V3 and clinical manifestations such as syncope, VA, and SCD. BrS prevalence is probably underestimated because the diagnosis requires a 12-lead ECG, and its first manifestation can be SCD. Reported prevalence ranges from 1 to 2000 to 1 to 5000 [165]. BrS is considered the cause of 10–20% of sudden infant deaths and 4–12% of SCD in children and young athletes [166], resulting in 20% of sudden unexplained death (SUD) in the young population [165]. Differently from the adult population where the incidence of the Brugada ECG pattern ranges from 0.12% to 0.8% [167,168], the prevalence in the pediatric population is likely underestimated because the most populous study in this subgroup of patients accounted for only thirty patients among thirteen tertiary hospitals in three different countries dating back 15 years [169]. The prevalence of the Brugada ECG pattern in asymptomatic children may vary between 0.01% to 0.02% based on large cohort studies conducted in Japan [170,171]. The Brugada pattern exists in children, and it becomes clinically relevant with age. This assumption is consistent with the fact that 23% of the asymptomatic children related to patients with Brs who tested negative on ajmaline challenge before puberty had abnormal ECG findings on ajmaline administration after puberty [172]. The clinical expression of Brugada disease typically starts in the fourth decade of life, but many malignant cases of VAs, sudden infant cardiac death, and SCD have been reported during childhood. The incidence of lethal arrhythmias due to BrS in children is around 10% [169].

### 6.2. Diagnosis and Electrocardiographic Pattern

**Figure 2:**
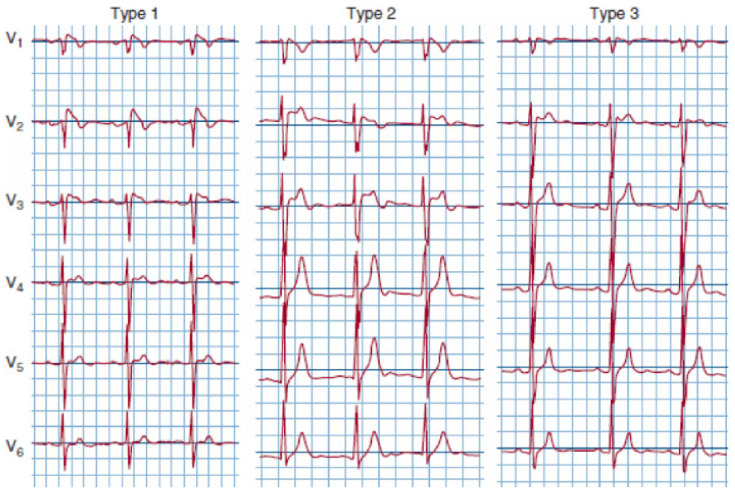
ECG showing different Brugada patterns.

It is necessary to distinguish between the diagnosis of the Brugada pattern based on ECG findings and BrS, which needs symptoms, in addition to ECG. The Brugada pattern is characterized by three different forms of ECG abnormalities (Figure 2). Brugada type 1 is associated with ≥2 mm ST-segment elevation with typical ST-segment coved appearance; BrS type 2 is characterized by ≥2 mm J-point elevation with <1 mm saddleback ST-segment elevation in the right precordial leads; and BrS pattern 3 is distinguished by ≥2 mm J-point elevation with <1 mm saddleback ST-segment elevation in the right precordial leads. Notably, BrS ECG findings are dynamic: in patients diagnosed with BrS type 1, abnormal ECG findings can be found in only one-third of the time [173]. In addition to ST-segment abnormalities, in the pediatric population, other abnormal ECG findings can be represented by the prolongation of PR, QRS, and QTc intervals [169]. In BrS, a combination of inverse and rapid repolarization myocyte membrane gradients generate typical ST-segment elevation and T-wave inversion; Brugada patients’ ECG are also characterized by an elevated T peak–T end, which is a repolarization index associated with a higher risk of arrhythmias or SCD [174]. A BrS diagnosis requires typical ECG findings associated with clinical symptoms such as palpitation, syncope, ventricular tachycardia, and ventricular fibrillation.

### 6.3. Pathophysiology

The major theories regarding BrS-related arrhythmogenesis are the depolarization and the repolarization theories.

Depolarization theory: sodium channels are fundamental for both conduction velocity and the propagation of action potentials through the myocardium. Anything that affects sodium channel function, thus modifying action potentials, may determine abnormalities in conduction and enhanced arrhythmogenesis [175]. Patients with BrS and a mutation of SCN5A have a reduced inward current during phase 0; this leads to slower upstroke in phase 0 with a subsequent delayed formation of the action potential. Typically, the delayed potential is measured in the RVOT epicardium area [176]. This theory is supported by the elevated number of conduction delays typically found in these patients such as intra-atrial conduction delay, AV block, and bundle branch blocks.

In the pediatric population conduction, delays are particularly numerous compared to the adult population, as reported by Chockalingam’s study [177], in which 85% of children had delays in cardiac conduction.

Repolarization theory: according to this theory, BrS arrhythmias would be caused by the variable action potential shortening along the myocardium. Mutated SCN5A may antagonize the delayed and rapid inactivation of Na+ channels, with different repolarization outcomes [178]. On the one hand, the repolarization is prolonged because, at slow cardiac rates, anomalies in rapid inactivation produce a persistent Na+ current. On the other hand, the intermediate kinetic component of slow inactivation is enhanced, slowing Na+ channel restoration, thus reducing Na+ current and abbreviating at higher heart rates than the action potential duration. In BrS, the action potential duration differs from the epicardium, where it is shorter, and the endocardium, where it is longer; this could be the result of relevant stronger transient outward current in the epicardium (Ito). This phenomenon can be seen in the epicardium as a more evident loss of the dome-shaped action potential form. All of this can explain the arrhythmia mechanism as a reentry thanks to a phase 2 reentrant pathway. This specific phase 2 reentry requires electrotonic interactions and also the diffusion from epicardial sites with the action potential dome to sites where there is no dome [179,180].

### 6.4. Genetics

The typical mutation of BrS affects the SCN5A gene located on chromosome 3, encoding a cardiac sodium channel located in the cell membrane. This mutation is inherited in an AD fashion, with incomplete penetrance, and determines a loss of function of the α-subunit in the sodium channel during the early part of phase I of the action potential. The first association between the BrS and SC5NA mutation was demonstrated by Chen et al. in 1998 [181].

While a patent pathogenic gene variant of SCN5A can be found in just 20–25% of the adult population with BrS [5,182], the prevalence of a gene mutation in pediatric patients is higher, reaching 58.1% [183]; accordingly, the American College of Medical Genetics guidelines recommends genetic testing for SCN5A in every child diagnosed with BrS [184].

There are many possible mutations of SCN5A, SCN10A, and L-type calcium channels, for a total of 16 genes that have been associated with BrS [185,186].

Considering that BrS is usually diagnosed between the third and fourth decade of life, the majority of children obtain a Brugada diagnosis as a consequence of family screening [187].

The inheritance of sodium channel mutations leads to AD. Having a pathogenic mutation in the SCN5A gene is significantly associated with a more arrhythmogenic substrate in RVOT [188] and an increased risk of VA [189].

The predictive value of having an SCN5A mutation was recently evaluated by Pannone et al. [190]. In a single center court of 63 patients, 29 had an SCN5A mutation and 34 did not, and this court was followed for 125.9 ± 176.4 months. Although no deaths happened in the pediatric cohort, eight children with BrS (12.7%) experienced VA, corresponding to an annual rate of 1.2%. At survival analysis, BrS pediatric patients with gene mutations had lower VA-free survival during follow up compared with BrS pediatric patients without gene mutations (79.3% vs. 94.1%, log-rank *p* = 0.03). Interestingly, the majority of mutations affect the pore region of sodium channels. Notably, along with the positivity of gene mutation, other independent predictors of VA were spontaneous BrS I patterns, sinus node disfunctions, and a history of aborted SCD. Although this evidence of a relevant role played by genetics; only a few symptomatic BrS have positive genetic testing. Clearly, other mechanisms are relevant in the pathophysiology of BrS, such as anomalies in fatty acid metabolism, specifically the deficiency of medium chain acyl-CoA [191].

BrS may be considered an allele pathology because different mutations on the SCN5A gene can provoke a different disease, such as long QT syndrome 3. A gain of function of the α-subunit of sodium channel can provoke a gain of function of the sodium channel, thus resulting in QT prolongation. BrS can overlap with ARVC because both diseases can have mutations in the connexome with different possible phenotypes [192,193]. PKP2 is responsible for BrS and/or ARVC, considering that it is associated with a deficit of sodium current, has anomalies in desmosomal integrity and can be usually found in BrS [194,195].

### 6.5. Clinical Manifestation in Children

BrS clinical presentation in children may be heterogeneous, including palpitation, syncope, AF, VT, VF, SCD, and SID [196]. The most frequent first evidence of BrS is a positive family history (47%), casual ECG findings (25%), syncope (14%), and arrhythmias (13%), such as AF (10%)-aborted SD (1%) [189,190,191,192,193,194,195,196,197]. The majority of syncopal episodes happen at rest and can be precipitated by fever, as well as vaccination-related fever [169,177]. Fevers can produce an imbalance between depolarizing currents and depolarizing currents during the action potential’s first repolarization phase, leading to specific ECG abnormal patterns [193,194,198,199]. An SCN5A mutation can be easily discovered by familiar anamnesis and pediatric ECG, and Holter monitor screening can identify ECG abnormalities considering their high prevalence [200].

A total of 50% of the first symptom of BrS is syncope or SCD; the syncope can begin early in a child’s life, can be frequent and repetitive through the years, and typically happens with a fever or at rest. Notably, syncope and the rate of other BrS symptoms do not differ between genders before adolescence; this differs from the predominance of the symptoms in adult male patients, which are probably addressed by the effect of action potential shortening in the RV epicardium by testosterone, thus facilitating VA initiation [201].

A particular form of syncope is the so-called “breathholding spell”, which can be found in 5% of the general pediatric population; this manifestation, which can be related to syncope, in particular, if iterative, could be addressed by arrhythmias and BrS [202]. The incidence of SCD in children is 1.3 per 100.000 persons/year [203], and 50% of the first presentation of symptomatic BrS in children is either syncope and/or SCD. A typical and characteristic BrS manifestation in children is SIDS. This clinical entity is represented by the abrupt death of infants <1 year old [204]. Such clinical syndrome is thought to be the main determinant of deaths in children between 1 month and 1 year of life [205]; a post-mortem molecular analysis has found SCN5A mutations in 10–15% of cases of SCD [206]. Furthermore, sodium channel complex genes were mutated in 12% of infant death syndrome in a nationwide registry in Denmark [169].

### 6.6. Evaluation of Brugada Syndrome in the Pediatric Population

#### 6.6.1. Symptomatic Children

It is unusual that a child is the first family member to manifest BrS-associated symptoms. Typically, a newborn is already surrounded by a familiar anamnesis that is positive for Brugada. In the suspect of Brugada, if any clinical event or rhythm disturbance related to BrS is found, the first step to evaluate a patient in the pediatric age group is the baseline 12-lead ECG and all first-degree relatives. In the case of a negative basal ECG, a diagnostic net between the emergency department, the pediatrics, and the family should be created in order to obtain a 12-lead ECG during a fever episode. Another diagnostic tool particularly useful in the pediatric population is the Holter ECG, which is capable of identifying sinus node dysfunction or AV block in periods of the day characterized by reduced physical activity. A treadmill exercise test is also useful to reveal sinus node dysfunction, consisting of chronotropic incompetence.

The standard test to unveil the Brugada ECG pattern is the so-called “provocative test”, consisting of the administration of intravein sodium channel blockers in those already suspected of having Brugada; there is still no consensus on the appropriate age when it is appropriate to start performing this test. Drugs that can be used are Flecainide (2 mg/kg, max 150 mg in 10 min) and Ajmaline (1 mg/kg, max 50 mg in 5 min). Ajmaline would be the best option between the two drugs due to its shorter half-life compared to flecainide, which requires a longer period of monitoring after the termination of the test. During the test, ECG is recorded and evaluated at the baseline and 1 min intervals during all test duration. In addition to drug administration, precordial leads V1 and V2 can be moved one intercostal space up from the usual fourth space in order to enhance the sensitivity of the test. The administration of these drugs in patients with Brugada that is not diagnosed can initiate VAs, and this is particularly frequent in symptomatic children or patients with conduction delays [207,208]. The provocative test is positive when the J-point elevation is >2 mV with coved ST-elevation in at least two precordial leads [209]. The next step in pediatric evaluation, when the above-mentioned tests were inconclusive, is the endocavitary EP study. With this test it is possible to assess the conduction properties of the heart, measuring the AH and HV intervals, sinus node function, sinus node recovery time, and Wenkebach cycle length, comparing each measurement with pediatric standards [210]. This test can be also evaluated by the arrhythmias inducibility with atrial programmed stimulation and ventricular programmed stimulation in basal conditions and during isoproterenol infusion. Considering the positivity for the SCN5A mutation in one-third of Brugada patients, according to the latest ESC guidelines [211], genetic testing should be reserved for a proband with a family history of BrS, such as newborns with first-degree relatives with BrS and ECG anomalies. Any SCD in a child must be evaluated for every family member with molecular autopsy [212] and the decedent’s blood sample or hair for SCN5A and genes for LQT1, LQT2, and CPVT [213]. Familiar anamnesis should be performed, including at least three generations and considering any episodes of previous recurrent syncope, sudden or premature death, epilepsy, drowning, car accidents, and epilepsy; it is fundamental to investigate the events that happened before the death in order to confirm its arrhythmic nature.

#### 6.6.2. Asymptomatic Children

The management of asymptomatic children belonging to a known Brugada family is still a matter of debate. A BrS investigation should be carried out in a family with at least one family member with BrS and should include a physical examination and a 12-lead basal ECG, especially during a fever episode. If there is a specific malignant family history, such as SCD or VAs, EPS can be considered in order to stratify the risk. The prognostic impact of EPS is still controversial. In the SABRUS study [214], the inducibility of VF via a programmed ventricular stimulation has been correlated with a similar time to the first arrhythmic event in a subject with a family history of SCD and SC5NA and longer time if compared to spontaneous type 1 ECG or syncope [215]. On the contrary, both FINGER [216] and PRELUDE [217] registries had an EPS that was negative for inducible VAs and was not linked to an inferior risk of arrhythmias. Also, the positive predictive value of EPS is under debate, considering that many studies [218] have demonstrated that managing to induce VT/VF predicts a higher risk of future arrhythmias, while other studies have proven the contrary [216,219].

### 6.7. Treatment

Drug therapy in children is limited to quinidine because lidocaine, mexiletine, beta-blockers, and magnesium have not been proven to be effective in this category of patients [220]. Quinidine is an inhibitor of the transient outward potassium current, managing to reduce the notch in the action potential and consequently suppress both the substrate and trigger for VAs [220]. The other therapeutic option available for children diagnosed with BrS with a proven efficacy in reducing SCD and sustaining VAs is the ICD implantation [221], which has a class I indication for BrS with past CA, spontaneous sustained VT with or without syncope, and class IIa in patients with spontaneous type 1 and syncope that can be likely provoked by VAs. Currently, there are no specific guidelines for device implantation in children affected with Brugada syndrome. The decision on the approach between transvenous, subcutaneous, or epicardial depends on the age of the patient, the level of physical activity, and body anthropometric characteristics. Dual chamber ICDs are reserved for those children with known SVT or SND. The main issue regarding ICD implantation in young patients lies in the high incidence of device-related complications such as lead fractures, inappropriate shocks, and the need for early reoperation or multiple operations in order to change ICD batteries [222]. Two aspects that must be considered with ICDs in young patients are reserving an extra lead length forming a loop in the right atrium in order to follow the child’s growth and avoiding dislodgment in case of a transvenous ICD; programming the ICD with the VT and VF zone set very high (>220 bpm), having a long detection interval (>30 s), and the activation of SVT discriminators, avoiding inappropriate shock for sinus tachycardia. Every ICD must be tested in the operating room with 10 J shocks in patients <25 kg and 15 J shocks for those >25 kg [223]. AF episodes in children are usually self-limiting [224], and they can be regarded as hints for BrS [196].

### 6.8. Follow Up

A Brugada diagnosis in a child comes with a great magnitude of suffering and anxiety. A multidisciplinary approach should be thought of for young BrS patients, including a pediatric cardiologist, a pediatric electrophysiologist, the family’s primary care physician, the geneticist, and the support psychologist. The fundamental aspect of the life of a young Brugada patient is the aggressive approach during fever episodes, treating them with a combination of acetaminophen and ibuprofen, as recommended by guidelines [225]. Antipyretics should be given to infants before routine vaccinations. Moreover, if any arrhythmia or ST-coved elevation should ever occur during the fever, the child should be monitored in the hospital during the fever period. Eventually, parents and children must be informed about all medications and drugs associated with increased risk of adverse outcomes in BrS, thanks to the list on the website www.brugadadrugs.orgs.org accessed on 20 September 2023.

## 7. Catecholaminergic Polymorphic Ventricular Tachycardia (CPVT)

This is a rare and dangerous channelopathy that can cause SCD, especially in the pediatric population. It is characterized by normal ECG at rest and the induction of bidirectional or polymorphic VT under adrenergic stress, both physical and emotional [226]. This disease is caused by a mutation in the channels and proteins responsible for the homeostasis of intracellular calcium.

### 7.1. Epidemiology

The true prevalence of this channelopathy is difficult to establish because the first manifestation is usually SCD at a young age in patients with de novo mutations, accounting for approximately 50% of total cases [227,228]. The estimated prevalence is 1 per 10,000, but it can be higher, as can be seen from the higher incidence of RYR2 mutation in the pediatric population affected by SCD [229,230,231]. The disease mainly presents at a young age, with an average age outset between 9 and 11 years; rarely, subjects develop the first symptoms in adulthood [232,233].

### 7.2. Pathophysiology

The mutations responsible for this disease encode intracellular proteins implicated in the homeostasis of intracellular calcium; intracellular calcium is indeed fundamental in the electromechanical coupling of the cardiac cell. Briefly, the propagation of the action potential in cardiomyocytes determines the opening of the L calcium channels, located on the T tubules, and the subsequent increase in the cytoplasmatic calcium. This determines the release of calcium from the sarcoplasmic tissue via RyR2 channels; this process is called Ca^2+^-induced Ca^2+^ release (CICR) [234]. The activity of the RyR2 channel is regulated by different molecules; one of these, CASQ2, a protein located within the sarcoplasmic reticulum that binds Ca with a high capacity and low affinity and regulates the level of free Ca^2+^ [235]. Once the cardiac depolarization phase is over, the excess of cytoplasmatic calcium is reabsorbed within the sarcoplasmic reticulum (SR) by the SERCA2a pump and partly extruded into the extracellular space by the Na/Ca exchanger (NCX). An overload of calcium within the SR can determine the spontaneous leakage of calcium into the cytosol, and through the NCX exchanger, produce an oscillation of the action potential and generate a delayed afterdepolarization (DAD), triggering arrhythmias [236,237,238].

The entire cycle is regulated by the sympathetic nervous system through the activation of protein kinase A (PKA), whose role is, through phosphorylation, to modulate and increase the activity of various proteins involved in this system, such as L-type Ca channels, RYR2, and phospholamban (PLB). The latter boosts the reuptake of calcium into the SR by disinhibiting the SERCA2a pump [239,240,241].

The main mutations involved concern two genes, RyR2, characterized by AD inheritance and responsible for at least half of the cases, and CASQ2 with AR inheritance, responsible for a much smaller portion of cases, approximately 2–5% [242,243]. The mutation of the RyR2 gene determines a gain of function of the channel, which becomes more prone to the spontaneous release of Ca^2+^ from the SR in diastole; this effect is further enhanced by the activation of the sympathetic system and, therefore, by a greater tendency to produce arrhythmias under stress [244]. Cases of RYR2 mutations with AR inheritance have also been described [245]. However, the mutation of the CASQ2 gene determines a lower binding capacity of the protein for Ca^2+^, with a greater concentration of free Ca^2+^ in the SR and, therefore, a greater tendency of spontaneous leakage of calcium into the cytoplasmic space [246].

Mutations in additional genes have emerged as possible causes of this syndrome [247,248]. In particular, using a framework based on the possible genetic and clinical correlations described in the literature, Walsh et al. confirmed a causal role for other genes in addition to the two previously reported: TRDN, which encodes for triadin, a protein that mediates the relationship between RYR2 and CASQ2; TECRL, which encodes for trans-2,3-enoyl-CoA reductase-like, a protein expressed in the endoplasmic reticulum of myocardial cells that plays a role in intracellular Ca^2+^ homeostasis; and three calmodulin genes (CALM1, CALM2, CALM3), which are located on different chromosomes but encode identical Ca binding proteins [249]. A recent investigation has shown how the pathologies caused by the calmodulin mutation had a phenotype not limited to the CPVT syndrome alone and are responsible for some forms of LQTS, with possible mixed phenotypes between these two syndromes, and for more complex clinical pictures that are characterized, in addition to arrhythmogenic manifestations, by neurological symptoms [250]. The penetrance and expressivity of all the genetic variants associated with CPVT are variable, explaining the different severity of the pathology. The specific tissue from which the arrhythmias originate is not clear yet. The ventricular ectopic beats that are first highlighted on the exercise test seem to originate from the RVOT. It is thought that both the conduction system, via the Purkinje fibers, and the ventricular working myocardium, are implicated with the genesis of the VT that is typical for this syndrome [247].

### 7.3. Diagnosis

As described in the international consensus statement in 2013, led by Priori et al. [221] at least one of the following criteria is required to diagnose CPVT:

1. The presence of a structurally normal heart, a normal ECG, and unexplained exercise or catecholamine-induced bidirectional VT or polymorphic ventricular premature beats or VT in an individual <40 years of age.
2. A patient (index case or family member) with a pathogenic mutation.
3. The family members of a CPVT index case with a normal heart who manifest exercise-induced PVCs or bidirectional/polymorphic VT. Instead, the diagnosis of CPVT can be made, with a lower degree of evidence, in the presence of a structurally normal heart and coronary arteries, a normal ECG, and unexplained exercise or catecholamine-induced bidirectional VT or polymorphic ventricular premature beats or VT in an individual >40 years of age [221]. Most patients have a normal ECG and do not have any structural alterations on echocardiogram or second-level imaging tests; the only resting ECG abnormalities reported are sinus bradycardia and the prominence of U waves [232,251,252,253]. The exercise treadmill test is the gold standard for the diagnosis of CPVT. This test typically is characterized, before reaching the target frequency, by ventricular extrasystoles, which are initially isolated and gradually, with the increase in heart rate, progress in complexity (multifocal, bidirectional, organized in pairs) until the genesis of monomorphic or bidirectional VT occurs, recognized as the signature arrhythmias of CPVT [254]. However, this test cannot obtain a 100% sensibility. For this reason, it may be useful to perform ambulatory ECG monitoring, a new exercise treadmill test over the years, or pharmacological testing with isoproterenol infusion, the latter used especially in children who are too young to perform an exercise test [255,256,257]. Genetic testing is important to confirm the diagnosis and start family screening, but its negativity should not exclude possible CPVT. In suspected cases, it is recommended to carry out genetic testing to look for mutations in the RYR2 and CASQ2 genes and not in broader genetic panels; instead, in familiar screening, the specific variant of the probands is looked for [213].

### 7.4. Clinical Manifestations

The first manifestation of the disease occurs predominantly before adulthood; rarely, the first symptoms are described in infants and adults [232,233,253]. Syncope is the most frequent first manifestation, although the disease often manifests itself with an aborted CA or SCD. Often, the diagnosis is delayed for about 2 years from the onset of symptoms, interpreting the events as vasovagal syncope, epilepsy, or a manifestation of LQTS with normal QT [227,258]. Patients frequently have a family history of SCD before the age of 40 [227,259]. The development of supraventricular arrhythmias, such as AF, AFL, or junctional tachycardia, is not unusual in these patients [256,260]. Several predictors of the severity of the disease have been described over the years, including the early onset of symptoms, the male sex, a history of syncope at onset, proband status, chronotropic insufficiency on exercise testing, RYR2 mutation, and which domain of channel is affected; however, none of these criteria found widespread consensus in the scientific community [227,232,233,256,261,262,263]. The 2022 European guidelines on the prevention of SCD, based on the work of Hayashi et al., report SCD risk markers in the diagnosis in childhood, the lack of beta-blocker therapy, and complex arrhythmias during the exercise stress test on a full dose of beta-blockers [244].

### 7.5. Therapy

The cornerstones of CPVT management are exercise restriction and beta-blockers without intrinsic sympathomimetic activity [25]. Non-selective beta-blockers, like nadolol and propranolol, are the most effective agents to prevent breakthrough arrhythmia in CPVT [262,264,265]. Also, genetically positive family members must be treated with beta-blockers, as recommended by European guidelines [211,221,256]. In addition to beta-blocker therapy or alternatively, when the burden of arrhythmias is not under control despite the maximum tolerated dose of these drugs or the patients do not tolerate them, flecainide can be used [266,267,268,269]. According to European guidelines, an ICD is indicated in the case of a patient resuscitated from CA, while it should be considered if the patient experiences significant VA despite optimized medical therapies [211]. However, the risk-benefit ratio of the defibrillator and its positive impact on survival are debated, especially if we consider that it is a young population. Complications related to the ICD system, including inappropriate shocks, are frequent. Recent evidence suggests that the implantation of an ICD does not lead to a significant increase in survival and, in addition, that a small percentage (1.4%) of patients is not protected from SCD despite the ICD [270,271,272,273]. These results may be related to the strong adrenergic stress determined by ICD shock, which can trigger the recurrence and persistence of VA, which can degenerate into an electrical storm. On the contrary, Mazzanti et al.’s group did not highlight any SCD in patients with an ICD and showed a significant impact on survival [262]. A further therapeutic option is left cardiac sympathetic denervation (LCSD). It has been found to be very effective in reducing potentially lethal arrhythmic events and appropriate defibrillator interventions if performed at experienced centers [274,275,276]. Therefore, LCSD should be considered when optimized medical therapy cannot be performed, or if its results are ineffective [211].

Based on these data and the guidelines, the ICD implant is recommended in secondary prevention of SCD; instead, in primary prevention, LCSD can be considered as an alternative or an addiction to the ICD. If one decides to proceed with the implantation of an ICD, it is essential to carefully program the device’s therapies, extending detection times and increasing cut-off rates to allow the self-resolution of the arrhythmias and minimize shocks to the patient. As a matter of fact, bidirectional VT is not due to reentry, and it is not treated by electrical therapies (ATP, shocks); electrical therapies can treat only VF [272,273]. The 2021 pediatric guidelines, in selected cases, propose LCSD and/or medical therapy to replace ICD implantation in aborted CA patients [277].

## 8. Congenital Long QT Syndrome

Congenital LQTS is a heterogeneous heritable condition characterized by a prolonged QT interval due to cardiac repolarization anomalies, recurrent syncope, and SCD [278]. Rare variants in 17 genes have been associated with the syndrome, with different degrees of penetrance and phenotypic expressions [278]. However, most cases are associated with AD mutations of KCNQ1, KCNH2, and SCN5A, responsible for LQTS1, LQTS2, and LQTS3, respectively. SCD risk stratification (based on ECG, clinical features, and genotype) is essential to allow the adoption of effective preventive and treatment measures.

### 8.1. Epidemiology

The estimated prevalence of LQTS is 1:2000, and it has a slight female prevalence [279]. The annual rate of SCD is 0.5% in the LQTS population, whereas the rate increases to 5% in patients with syncope history. Almost 15% of unexplained resuscitated CA and 5% to 10% of young SCD referred for autopsy are related to LQTS [280,281].

### 8.2. Pathophysiology

Loss-of-function in voltage-gated potassium channels is responsible for the majority of LQTS, with a reduction in the outward potassium current during phase 3 of the action potential. LQTS1 is caused by a mutation in KCNQ1, encoding for the α-subunit of Kv7.1, and LQTS2 is associated with a mutation in KCNH2, encoding for the α-subunit of Kv11.1 [282]. Mutations in β-subunits of the same channels are related to LQTS5 (KCNE1) and LQTS6 (KCNE2) [283]. An increased inward sodium current during the plateau phase of the action potential is related to a gain-of-function mutation in the SCN5A gene and is responsible for LQTS3 [284]. Overall, these mutations lead to the prolongation of action potential duration with the occurrence of early afterdepolarizations and torsade de pointes. Moreover, such mutations blunt the effect of adrenergic stimulation on depolarization and repolarization kinetics, resulting in increased QT variations associated with heart rate changes [285].

### 8.3. Diagnosis

The diagnosis of LQTS is based on the modified LQTS diagnostic criteria score, the “Schwartz score” [282]. As shown in Table 1, the diagnostic score is based on ECG, clinical, family, and genetic findings. LQTS is diagnosed in the presence of QTc ≥ 480 ms in repeated 12-lead ECGs with or without symptoms; a pathogenic mutation irrespective of QTc duration; and an LQTS score > 3. Moreover, LQTS diagnosis should be considered in the presence of QTc ranging from 460 and 480 ms associated with arrhythmic syncope in the absence of secondary causes of QTc prolongation [204].

#### 8.3.1. Clinical Manifestations

Recurrent syncope, associated with hemodynamically non-tolerated VA, is the classical manifestation of LQTS. Symptoms are triggered by different stimuli that are genotype-specific, such as exercise in LQTS1, emotional stress, the post-partum period or sudden auditory stimuli in LQTS2, and sleep in LQTS3 [206]. In addition, other manifestations are SCD, resuscitated CA, atrial arrhythmia, and epilepsy due to the sodium and potassium homeostasis derangement related to gene mutations.

#### 8.3.2. ECG Features

QTc prolongation is the landmark sign of LQTS. However, long QTc values may be recorded in up to 10% of the general population, whereas up to 40% of genotype-defined LQTS may display normal QTc. Hence, QTc duration cannot be considered as a standalone criterion for diagnosis but should be associated with clinical and genetic evaluation. The QT interval is measured from the beginning of QRS to the end of the T-wave, preferably in lead II or V5, using the tangent or threshold technique and excluding the U wave in the measurement. Of course, the QT interval should be corrected for heart rate using the Bazett formula [282]. Beyond the QTc interval, T-wave alternans and typical T-wave patterns, such as notched T-waves, may be present and indicate a particular underlying genotype [286].

#### 8.3.3. Provocation Testing

LQTS is characterized by a maladaptive repolarization response to autonomic changes, particularly during the acceleration and deceleration of heart rate [287]. Exercise testing allows for the useful assessment of repolarization response during heart rate variations, with a QTc interval ≥ 445 ms at 4 min recovery showing high sensitivity and specificity for LQTS diagnosis [288]. In addition, temporal changes in QTc associated with exercise or recovery may differentiate LQTS genotypes [289]. Importantly, in the pediatric population, a longer observational period and QTc interval is recommended and validated, with a QTc measurement > 460 ms obtained at 7 min recovery, suggesting LQTS diagnosis [289]. Abrupt standing from a supine position induces an immediate and long-lasting increase in the QTc interval in LQTS patients [290]. However, this measure is not validated in the pediatric population because increased QTc is also found in healthy children [289]. Currently, the epinephrine challenge is not routinely recommended, but it can be proposed for patients unable to perform exercise [204].

#### 8.3.4. Genetic Testing

Genetic testing yields a 75% probability of identifying a culprit genetic variant, with the three main genes accounting for up to 90% of positively genotyped cases [204,282]. KCNQ1 causes LQTS1 (40–45%), KCNH2 is related to LQTS2 (40%), and SCN5A is responsible for LQTS3 (5–10%). However, our knowledge of the genetics of LQTS is still growing with the discovery of LQTS susceptibility genes [291] and the recent identification of polygenic influences in LQTS patients [292].

### 8.4. Risk Stratification

Clinical, ECG, and genetic factors are associated with different risks of serious arrhythmic events (SAEs), such as SCD and resuscitated CA, and should be considered when deciding preventive and treatment measures in LQTS patients.

From a clinical standpoint, the occurrence of syncope confers an increased risk of SAE, which is greater in patients with recurrent and/or recent (<2 years) syncope [293]. Moreover, arrhythmic events are more common during childhood, and there is a higher incidence in males as compared to females until age 13 [294]. During adolescence, the incidence of SAE is similar among the sexes, whereas beyond 18 years, females have a higher relative risk of SAE and syncope compared to males [295]. These sex-related differences seem to be related to the sex hormone effect, with testosterone increasing repolarizing potassium current and estrogen inhibiting it [296].

Of note, QTc interval duration strongly influences the risk of arrhythmic events. Indeed, QTc ≥ 500 ms is related to a three-fold increased risk of arrhythmic events compared to QTc < 500 ms [294], and an increased relative risk for events of 1.1 has been described for each 10 ms QTc increment [297]. Moreover, Sauer et al. [295] found an association between QTc interval duration and the risk of arrhythmic events, with longer QTc intervals associated with higher event prevalence over time.

The LQTS genotype influences the risk of arrhythmic events. Indeed, the annual rate of SAE and syncope up to the age of 40 years differs among the three main genotypes: 0.8% to 1.1% in LQTS1, 0.8% to 1.8% in LQTS2, and 0.5% to 1.5% in LQTS3 [298]. In addition, the age of clinical presentation and increased mortality varies according to genotype: 1 to 19 years for LQTS1, 30 to 39 years for LQTS2, and 15 to 19 years for LQTS3 [299]. Notably, LQTS2 portends an increased risk of SAE in the post-partum period [300]. Current guidelines suggest the use of the 5-year 1-2-3 LQTS risk calculator to identify asymptomatic patients with a high risk of arrhythmic events (≥5% in 5 years) in whom prophylactic ICD therapy should be considered [204,301]. This risk score considers QTc interval duration and LQTS genotype. Mounting evidence suggests that genetic stratification extends beyond the LQTS subtype. Indeed, a strong impact of specific gene variants and their location within the potassium channel has been demonstrated, modifying the risk classically associated with each genotype [302,303]. Therefore, genetic stratification should include not only the genotype but also variant identification and interpretation to correctly stratify individual arrhythmic risk.

### 8.5. Management

#### 8.5.1. Conservative

All LQTS patients should follow the following non-pharmacological recommendations: avoid QT-prolonging drugs, avoid and correct electrolyte abnormalities, and avoid genotype-specific triggers for arrhythmia [204]. In addition, illicit substances should be avoided. Usually, these conservative measures are enough for asymptomatic patients without QTc prolongation. Currently, LQTS is not a contraindication to sport participation in patients adequately stratified and treated [304], with the only exception being swimming in LQTS1 patients due to the risk of drowning.

#### 8.5.2. Pharmacological

Non-selective beta-blockers are the mainstay of pharmacological therapy of LQTS. Beta-blockage leads to QTc and repolarization heterogeneity reduction, resulting in a 50–60% reduction in arrhythmic events [294,305]. Particularly, nadolol, at a dose of 1 mg/kg/die, and propranolol provide the greatest event risk reduction in LQTS1 and LQTS2 [306]. Beta-blockers are recommended in all LQTS patients with prolonged QT intervals to reduce arrhythmic events and should be considered in asymptomatic patients with normal QTc intervals [204]. Although evidence for beta-blockage in LQTS3 is less robust, mexiletine at a dose of 8 mg/kg/die reduced adverse events by counteracting the increased sodium current due to the pathogenic mutation of the SCN5A gene [307].

#### 8.5.3. Device Therapy

ICD therapy, on top of beta-blockers, is recommended in the secondary prevention of SCD in patients who experienced an aborted CA. An ICD is also indicated in patients with cardiogenic syncope and/or hemodynamically non-tolerated VA, despite pharmacological therapy [204]. A shared decision-making discussion on ICD implants should be considered in symptomatic LQTS patients in whom pharmacological therapy is contraindicated or not tolerated and may be considered in asymptomatic patients with a high risk of arrhythmia according to the 1-2-3 LQTS risk calculator [301,308]. Patients with Jarvell and Lange–Nielsen Syndrome (JLNS) are considered at a high risk of SCD, with 35% of patients dying by age 40 despite pharmacological therapy [309]. Those with QTc intervals greater than 550 ms, syncope before age 5, and males older than 20 years with the *KCNQ1* pathogenic variant have the worst prognosis and should receive an ICD [310]. Of note, in symptomatic pediatric patients with a high arrhythmic risk, the combination of beta-blockers and atrial pacing (without an ICD) was demonstrated to reduce adverse arrhythmic events [311].

#### 8.5.4. Left Cardiac Sympathetic Denervation (LCSD)

Autonomic modulation therapy is indicated in symptomatic patients, despite pharmacological and device therapy, or in symptomatic patients who are not willing to receive ICD therapy. In addition, LCSD should be considered as an alternative to ICD therapy in patients intolerant of beta-blockers [204]. LCSD using video-assisted thoracic surgery is associated with minimal perioperative complications and excellent short- and long-term outcomes [312].

## 9. Short QT Syndrome (SQTS)

SQTS is a rare, inherited channelopathy that is associated with cardiac arrhythmias and SCD. The syndrome is characterized by short QT and often clinical presentation in infants and is related to a mutation in eight genes regulating ionic currents, following an AD pattern of inheritance [313]. However, comprehensive genetic testing has a low diagnostic yield (20–30%), and the risk stratification of SQTS patients is still a matter of debate due to the low prevalence of the syndrome [314].

### 9.1. Epidemiology

SQTS prevalence differs among adult and pediatric populations, with European registries reporting a prevalence between 0.02 and 0.1% in adults and up to 0.05% in the pediatric population [315]. However, SQTS prevalence strongly depends on the QTc interval cut-off used, with a suggested value of ≤320 ms to avoid overdiagnosis [316]. Although arrhythmic events may occur at all ages, the rate of events is particularly high in the first year of life, accounting for up to 4% of SCD [317]. The condition is highly lethal, with a cumulative risk of cardiac events of 40% by 40 years and a male predominance, probably due to the effect of testosterone on ionic current [318,319]. A personal or familiar history of SCD is present in almost 80% of patients.

### 9.2. Pathophysiology

SQTS is related to gain-of-function mutations in genes encoding potassium outward currents and loss-of-function mutations in genes encoding calcium inward currents. The resulting shortening of action potential duration is heterogeneous, leading to the transmural dispersion of repolarization that predisposes it to reentrant arrhythmias, such as AF and VF [320].

### 9.3. Diagnosis

Current European guidelines [204] recommend SQTS diagnosis in the presence of QTc ≤ 360 ms and one or more of the following: a pathogenic mutation, a family history of SQTS, and survival from VT/VT. In addition, SQTS diagnosis should be considered in the presence of QTc ≤ 320 ms or QTc ≥ 320 ms and ≤360 ms and arrhythmic syncope. Eventually, SQTS may be considered in the presence of QTc ≥ 320 ms and ≤360 ms and a family history of SCD at age < 40 years. Gollob et al. proposed a diagnostic score for SQTS, including family history, ECG features, and gene mutations that are known to be associated with SQTS as diagnostic criteria (Table 2) [321].

### 9.4. Clinical Manifestations

Almost 40% of patients are asymptomatic, although SCD could be the first clinical manifestation. Commonly, SQTS is associated with palpitations, AF, VA, and syncope. SCD risk is higher during the first year of life and between 20 and 40 years [322].

### 9.5. ECG Features

Of note, QTc should be measured at a heart rate within normal limits because of the lack of rate dependence in patients with SQTS and the risk of overestimating QT when corrected for a higher heart rate [313]. Beyond the short QTc interval, SQTS is characterized by additional ECG alterations and features, such as absent or minimal ST-segments, tall T-waves, early repolarization patterns, U waves, and short intervals from the J-point to T-wave peak (J-T peak) [313]. Recently, Suzuki et al. reported novel ECG criteria for SQTS in children and adolescents [323]. In particular, in a population of 34 patients with a median age of 14 years (interquartile range 0–19), they found that a QT corrected by Bazett’s formula (QTcB) < 316 ms had 79.4% sensitivity and 96.7% specificity for SQTS diagnosis. Moreover, a J-T peak cB < 181 ms showed 80.8% sensitivity and 91.8% specificity for diagnosis. Eventually, Suzuki et al. [323] found a significantly higher prevalence of early repolarization patterns among SQTS patients compared to the control group (67% vs. 23%). Giustetto et al. reported that a QT/HR relationship slope under −0.9 ms/beat/min in an exercise test is highly predictive of SQTS [324]. Other proposed features are a reduced QT adaptation to standing and a PQ segment depression [325].

### 9.6. Genetic Testing

Genetic analysis allows for the identification of the potential damaging variant in almost 30% of cases [313]. Current guidelines recommend testing five genes in all patients diagnosed with SQTS and their first-degree family members: KCNH2, KCNJ2, KCNQ1, CACNA1C, and CACNB2B. KCNH2 is the most frequently involved gene, encoding a voltage-activated potassium channel and responsible for the so-called SQTS type 1 (15% of all cases) [326]. KCNQ mutations are associated with SQTS type 2, accounting for 5% of all cases, whereas KCNJ2 encoding for an inward-rectifier type potassium channel is related to SQTS type 3 (nearly 5% of all cases) [327,328]. SQTS types 4 and 5 are associated with mutations in genes encoding subunits of voltage-dependent calcium channels (CACNA1C and CACNB2B), which account for < 1% of all cases. Variants in genes of CACNA2D1 for a protein of the voltage-dependent calcium channel complex, SCN5A for the α-subunit of the sodium channel protein, and SLC4A3 for the plasma membrane anion exchange protein 3 have been related to SQTS, although no conclusive data exist regarding the association of these genes with the syndrome [313].

## 10. Risk Stratification and Management

The only predictor of SCD in SQTS patients is the history of a previous CA; hence, ICD therapy is recommended in survivors of an aborted CA [204]. In addition, patients with spontaneous documented VT should receive ICD therapy. Regarding primary prevention, the risk stratification of SQTS patients is challenging due to the lack of independent risk factors associated with SCD in asymptomatic patients. Counterintuitively, a shorter QT does not represent SCD risk makers [329], whereas a strong family history of SCD and a personal history of arrhythmic syncope have been associated with increased SCD risk [204]. As a result, current guidelines consider ICD implantation in SQTS patients with arrhythmic syncope. However, ICD therapy benefits should be balanced with the risk of inappropriate ICD therapies and device-related complications, especially in the pediatric population. In asymptomatic young patients, ILR should be considered for arrhythmia monitoring, as well as pharmacological therapy with QTc-prolonging drugs, such as quinidine [330]. Quinidine may be considered a chronic pharmacological treatment in SQTS patients with contraindications to ICDs who are not willing to receive an ICD and in asymptomatic SQTS patients with a family history of SCD. EPS is not recommended for risk stratification [204].

### 10.1. SCD Investigation in Young Patients and the Management of the Surviving Family

#### SCD

The diagnosis of SCD causes and the management of asymptomatic relatives of SCD victims are quite challenging and aims to establish if an inherited CM is present to protect the patient from SCD at-risk family members, facilitating the initiation of appropriate prevention strategies [5]. However, SCD is frequently the first presentation of the disease, and nearly 30% of SCD cases in the pediatric population remain without a clear cause of death after a full post-mortem forensic autopsy, including detailed macroscopic and histological heart evaluation [331]. In these cases, an inherited CM is highly suspected and probably the cause of death. In this scenario, genetic testing of the decedent’s blood sample, the so-called “molecular autopsy” is indicated, allowing for an explanation of the SCD cause in nearly 20% of cases and the identification of asymptomatic genetic carriers.

In recent years, the molecular diagnosis of an inherited CM has been facilitated by the advancement of next-generation sequencing (NGS) technology. NGS has arisen from DNA sequencing methodologies and can be applied to the diagnosis of inherited diseases in three ways: targeted sequencing for a number of genes (multigene panels), whole-exome sequencing (WES), and whole-genome sequencing (WGS). It has been recommended that the most cost-effective first-line testing is gene panels or exome-sequencing-based analysis of genes, allowing a cost- and time-effective approach to genetic analysis [332].

Concurrent with molecular autopsy, clinical investigation of the victim’s relatives is important, as it may reveal the disease in the family. Indeed, 95% of the inherited CMs follow an autosomal-dominant pattern, and 50% of first-degree relatives present the index pathogenic mutation. Hence, all asymptomatic relatives should have a comprehensive clinical and family history, physical examination, resting and exercise ECG, and an echocardiogram. Furthermore, if a specific CM is suspected, cardiac magnetic resonance, 24 h ECG monitoring, and pharmacological challenge tests may be performed [212]. If molecular autopsy results in a genetic diagnosis of the decedent, genetic testing is initially performed on the parents to determine if the mutation does not arise de novo and, subsequently, cascade genetic screening should be performed on at-risk family members, together with pre- and post-test genetic counseling [212]. After the genetic diagnosis is made, the management of relatives will depend on the specific disease and mutation. If no diagnosis is made after a genetic test, children are periodically followed up until adulthood (up to 40 years), whereas asymptomatic adults are discharged after age 40 years, considering that most inherited CMs are phenotypically expressed during the first four decades of life.

## Figures and Tables

**Table 1:** Schwartz score.

ECG features *
A QTc interval ^#^, ms ≥480 3 460–479 2 450–459 (males) 1
B QTc interval ^#^ ≥ 480 ms at 4 min recovery from the exercise stress test 1
C Torsade de pointes ^§^ 2
D T-wave alternans 1
E Notched T-wave in three leads 1
F Low heart rate for age ^δ^ 0.5
Clinical history
A Syncope ^§^ With stress 2 Without stress 1
B Congenital deafness 0.5
Family history ^&^
A Family member with definite LQTS 1
B Unexplained sudden cardiac death age <30 years among immediate family members 0.5

Score ≤ 1: low probability; 1.5 to 3, intermediate probability; ≥3.5, high probability. * In the absence of medications or disorders known to prolong QTc. ^#^ QTc interval calculated by the Bazett formula. ^§^ Mutually exclusive. ^δ^ Resting HR < second percentile for age. ^&^ The same family member cannot be used for both A and B. LQTS: long QT syndrome.

**Table 2:** SQTS diagnostic criteria (a score of ≥ 3 indicates a moderate-to-high probability of SQTS).

SQTS Diagnostic Criteria	Points
*QTc interval (ms)*	
<370	1
<350	2
<330	3
*J-point-to-T peak interval < 120 ms*	1
Family history *	
SQTS high-probability in a first- or second-degree relative	2
Autopsy-negative SCD in a first- or second-degree relative	1
SIDS	1
*Genotype*	
Genotype positive	2
Mutation of undetermined significance in a culprit gene	1

* ECG should be recorded in the absence of medications or disorders known to prolong QTc. SIDS: sudden infant death syndrome; SQTS: short QT syndrome.

## Data Availability

Not applicable.

## Abbreviations

**ACMs** = arrhythmogenic cardiomyopathies; **AD** = autosomal dominant; **AF** = atrial fibrillation; **ALCAPA** = Anomalous left coronary artery from the pulmonary artery; **ALVC** = arrhythmogenic left ventricular cardiomyopathy; **AR** = autosomal recessive; **ARVC** = arrhythmogenic right ventricular cardiomyopathy; **ASD** = aborted sudden death; **BrS** = Brugada syndrome; **CA** = cardiac arrest; **CAD** = coronary artery disease; **CHD** = congenital heart disease; **CM** = cardiomyopathy; **CMR** = cardiac magnetic resonance; **CPVT** = catecholaminergic polymorphic ventricular tachycardia; **DCM** = dilated cardiomyopathy; **DSP** = desmoplakin; **EMB** = endomyocardial biopsy; **EP** = electrophysiological; **HCM** = hypertrophic cardiomyopathy; **HF** = heart failure; **IA** = inherited arrhythmia; **LBBB** = left bundle branch block; **LGE** = late gadolinium enhancement; **LQTS** = long QT syndrome; **LV** = left ventricular; **NDLVC** = non-dilatated left ventricular cardiomyopathy; **PVCs** = premature ventricular contractions; **RBBB** = right bundle branch block; **RV** = right ventricular; **RVOT** = right ventricular outflow tract; **SCD** = sudden cardiac death; **SHD** = structural heart disease; **SQTS** = short QT syndrome; **TFC** = task force criteria; **VA** = ventricular arrhythmias; **VEBs** = ventricular ectopic beats; **VF** = ventricular fibrillation; **VHD** = valvular heart disease; **VT** = ventricular tachycardias
